# The impact of statutory net-zero targets on clean energy investment

**DOI:** 10.1016/j.isci.2026.117647

**Published:** 2026-09-25

**Authors:** Xiao Yan Zhou, Min Ruan, Jing Yu, Sam Fankhauser, Gireesh Shrimali

**Affiliations:** 1 The Smith School of Enterprise and the Environment, University of Oxford, Oxford OX1 3QY, UK; 2 Business School, The University of Sydney, Sydney, Australia

**Keywords:** national net-zero legislation, capital expenditure, low-carbon energy, corporate net-zero transition disclosure

## Abstract

Setting national targets for reaching net zero is a key indicator of countries’ commitment to climate action. But how effective are net-zero targets in driving down emissions? Here, we use a difference-in-differences model to study the link between statutory net-zero targets in 24 countries and clean energy investment from 2014 to 2023. We find that laws with target years of 2045 or earlier are associated with economically large and statistically significant increases in low-carbon investment, whereas laws targeting 2050 or later show no comparable average effect without frequent NZT reporting. Investment responses are stronger among firms with higher net-zero disclosure scores and in countries that report progress toward net-zero targets more frequently. These findings suggest that legislative ambition and transparency matter. Only credible and ambitious net-zero targets are able to mobilize private low-carbon investment.

## Highlights

• Statutory net-zero targets of 2045 or earlier increase clean energy investment
• Later statutory targets show no comparable effect without frequent national reporting
• National net-zero reporting and corporate disclosure enhance investment responses

## Graphical abstract

**Figure:**
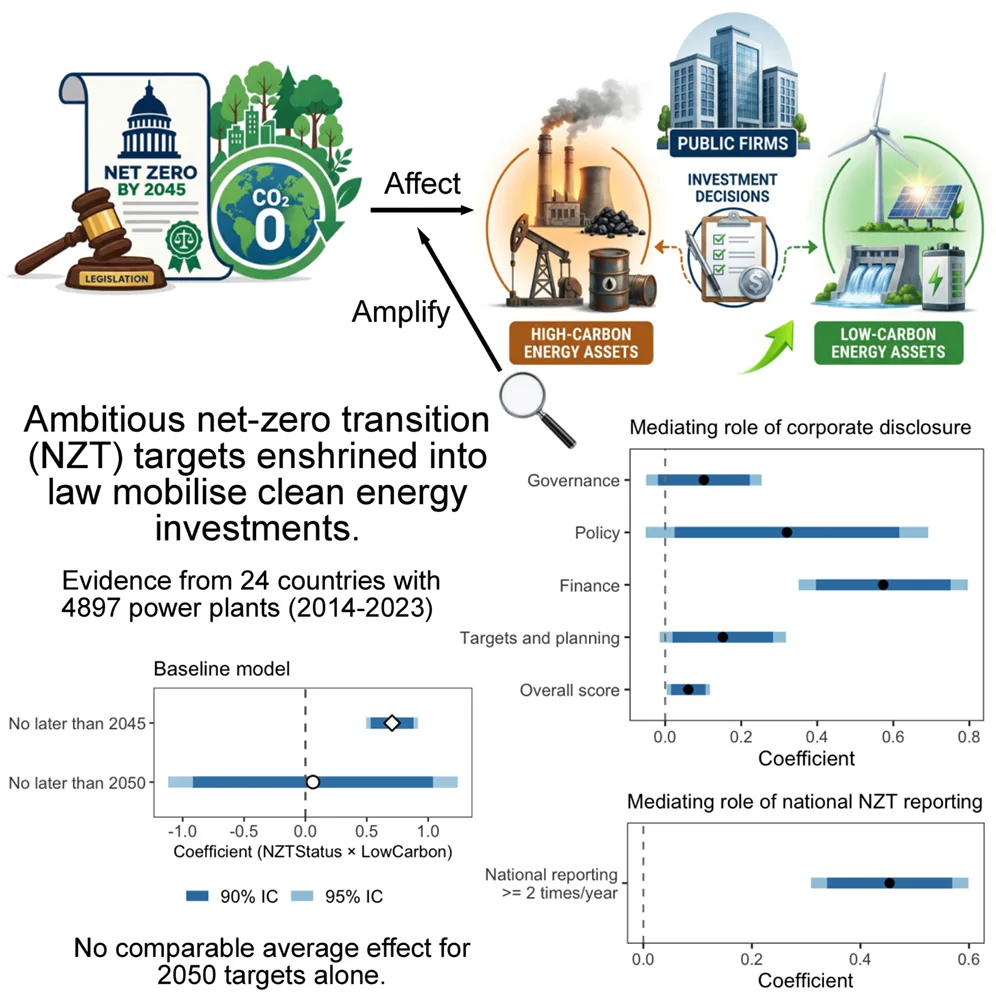

## Teaser

Environmental policy; Environmental technology; Energy policy

## Introduction

Countries have formalized their commitment to reducing greenhouse gas (GHG) emissions and achieving net-zero targets primarily through nationally determined contributions (NDCs) under the Paris Agreement or domestic climate policies.^1^ The anticipated benefits of net-zero legislation are manifold. They can establish the legal framework for public agencies to implement diverse climate mitigation and adaptation policies.^2^^,^^3^^,^^4^ Hence, these new laws offer policy certainty to reduce regulation transition risk for renewables^5^^,^^6^ and help drive investments in low-carbon technologies.^7^^,^^8^ Additionally, they may foster heightened awareness of a social challenge among key stakeholders,^9^ including companies, investors, and the general public,^10^ thereby incentivizing climate-friendly investments and consumption activities.^11^ Recent syntheses of ex post evidence suggest that mitigation policies have had measurable impacts on emissions and the deployment of low-carbon technologies, while highlighting substantial variation across instruments, jurisdictions, and implementation contexts.^12^

Yet doubts remain about how effectively these climate laws will be implemented, whether these climate pledges are truly credible, and whether countries will deliver on their commitments at the micro level.^13^ Specifically, there is a lack of empirical evidence on the micro-economic impact of different legal statuses of national net-zero transition (NZT) targets. It is thus important to understand whether and how the national NZT legislation effectively accelerates corporate decarbonization processes.^14^ One potential mechanism through which national NZT legislation influences corporate climate action is that legitimacy pressures may drive firm-level alignment behavior,^14^^,^^15^^,^^16^ including but not limited to enhancing sustainability disclosure^17^ and increasing investment in low-carbon energy.

Our analysis starts with a full sample of global energy assets in the power sector across the period of 1990–2030. To get an overview of low-carbon investments worldwide, we identify a subsample of assets owned by publicly listed firms, as the financial characteristics and ownership data for private firms are missing. We compare two samples in terms of (1) geographic location of high-carbon versus low-carbon assets, (2) changes in asset counts and installed capacity by energy type, and (3) the ratio of low-carbon to high-carbon asset counts and the ratio of low-carbon to high-carbon installed capacity over time, to understand the global reallocation and new deployment of pollutive and clean energy assets between public and private firms. The data pattern shows that publicly listed firms in our sample hold a higher share of low-carbon assets than the global average. The ratio differences between public and private firms have steadily increased since 2020, when the European Green Deal was approved, and many large firms announced their NZT targets.

We then merge data from different sources and construct a novel multi-level dataset encompassing asset-level capital expenditures, firm-level NZT disclosures, and financial characteristics, as well as country-level legal and economic indicators. Using this dataset, we systematically examine how the legal status and ambition of national NZT legislation relate to capital allocation between high-carbon and low-carbon energy assets among publicly listed firms. We find statistically significant increases in low-carbon capital expenditure following the enactment of NZT laws only in the subgroup of countries with statutory targets of 2045 or earlier; the estimates remain stable across pre-trend checks and robustness tests. Our research provides large-scale evidence on how different forms of national NZT legislation are associated with corporate investment behavior.

To investigate the mechanism through which the national NZT legislation affects public firms’ allocation of capital to low-carbon energy assets, we examine the roles of the corporate NZT score measured by climate-related disclosure at the firm level and the national NZT reporting frequency at the country level. The findings show that NZT legislation may make countries report progress at least twice per year and push public firms to disclose more information on emission targets and planning, financial support, and policy implementation, and thus drive alignment behaviors including increased low-carbon energy investments. This indicates that the positive effects of NZT legislation are reinforced by transparent and credible firm-level sustainability disclosures and frequent country-level climate transition progress reporting.

## Results

### Publicly listed firms hold a larger share of low-carbon assets

The global power asset-level dataset provides valuable information about capacity, energy type, start year of operation, geographic location, and company owner. We shed light on the full sample of 250,626 global energy assets in the power sector and identify a subsample of 26,275 assets owned by publicly listed firms worldwide.

In the full sample, high-carbon (i.e., fossil fuels) assets account for approximately 70.8% of total assets and low-carbon (i.e., renewables and storage) assets represent around 29.2% (Figures 1A and 1B). The spatial pattern in the subsample broadly mirrors that of the overall sample but with a comparatively higher concentration in developed economies, particularly North America, Europe, and East Asia (Figures 1C and 1D). The proportion of low-carbon assets owned by publicly listed firms is about 84.5% (Figure 1D), 13.7% higher than the global average level (Figure 1B). Comparing the geographic distribution of power assets in two samples, we find that those fossil fuel assets located near the Arctic Circle and offshore are primarily owned by private firms. This pattern is consistent with publicly listed firms holding a larger share of low-carbon assets than private firms.

**Figure 1:**
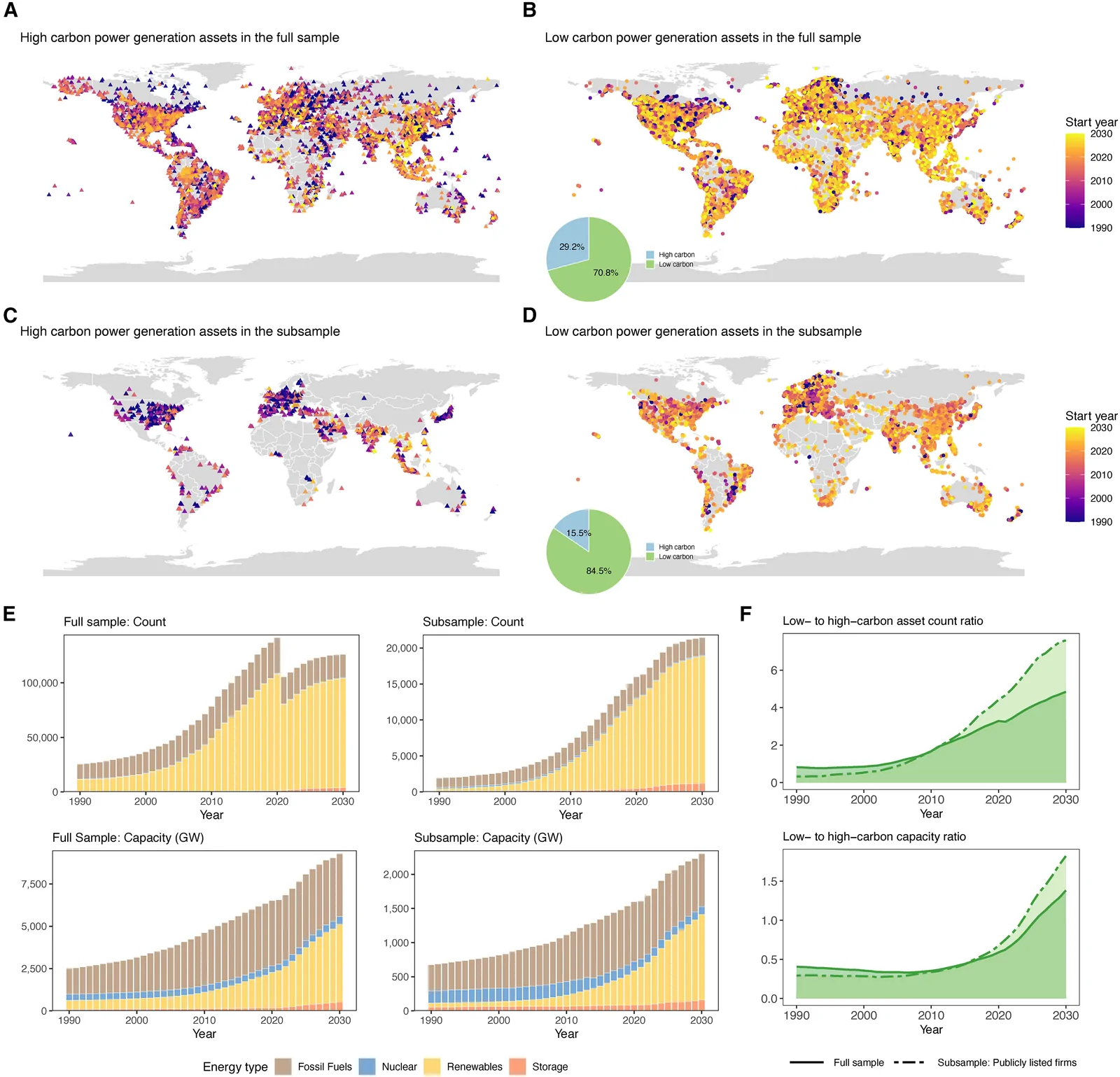
Visualization of global energy assets in the power sector (A) The geographic distribution of global high-carbon (fossil fuels) power generation assets (full sample) colored by start year of operation. (B) The geographic distribution of global low-carbon (renewables and storage) power generation assets (full sample) colored by start year of operation, with the proportion of high-carbon and low-carbon assets in the full sample. (C) The geographic distribution of global high-carbon power generation assets owned by publicly listed firms (subsample) colored by start year of operation. (D) The geographic distribution of global low-carbon power generation assets owned by publicly listed firms (subsample) colored by start year of operation, with the proportion of high-carbon and low-carbon assets owned by publicly listed firms in the subsample. (E) The number and installed capacity of global power generation assets and power generation assets owned by publicly listed firms, by energy type (fossil fuels, nuclear, renewables, and storage) and by year. (F) The ratio of low-carbon to high-carbon asset counts and the ratio of low-carbon to high-carbon installed capacity in the full sample (solid line) and in the subsample (dashed line). Assets with start years before 1990 are colored the same as those starting in 1990.

Compared with low-carbon assets, the number and installed capacity of fossil fuel assets continue to increase in absolute terms but at a slower rate (Figure 1E). Nuclear and storage assets remain very limited, yet the number and installed capacity of storage assets are expected to grow steadily between 2020 and 2030 (Figure 1E). The number of fossil fuel assets is relatively low in the full sample and subsample, while the aggregated installed capacity of these is considerable as most renewable projects are relatively small-scale (Figure 1E).

The preference for low-carbon assets among public firms was growing over time. The low-to high-carbon asset count ratio and installed capacity ratios of public firms both exceeded the global average in the 2010s, and the divergence has been widened since 2020, coinciding with the period when many countries announced their NZT targets through NDCs under the Paris Agreement (Figure 1F).

### Ambitious legally binding targets increase low-carbon investment

The regression analyses take advantage of the quasi-natural experiment created by the national NZT legislation across 24 countries. These countries announced their NZT targets between 2019 and 2021; Among them, 14 countries have legislated NZT targets, while the remaining countries have adopted them through policy documents, declarations, or proposals, with target years ranging from 2030 to 2070; interim commitments also vary, ranging from emission reduction targets to emission intensity and business-as-usual reduction targets, reflecting different levels of ambition and decarbonization pathways (Table 1). Based on the baseline DID model in Equation 1 (see STAR Methods), we estimate the national NZT legislation effects on corporate capital expenditure on low-carbon energy assets (Table 2). Columns (1)–(3) report the results using NZTStatus1, which identifies countries with statutory NZT targets of 2050 or earlier, while columns (4)–(6) report the results using NZTStatus2, which identifies the subset of countries with more ambitious statutory NZT targets of 2045 or earlier. The preferred estimation results contained in columns (3) and (6) control for country, year, and energy type fixed effects. In the preferred model (column 6), the coefficient of 0.706 implies that capital expenditure on low-carbon energy assets is approximately 103% ([*e*^0.706^−1] ×100 = 103%) higher following the enactment of a statutory NZT law with a target year of 2045 or earlier. This finding indicates that legislative ambition, in addition to legal status, is an important factor associated with stronger low-carbon investment responses. In contrast, the coefficient on NZTStatus1 × LowCarbon is statistically insignificant across all specifications, suggesting that the average association across this broader group is relatively weak and heterogeneous. To further substantiate this observation, we conduct a subsample analysis using only low-carbon energy assets and control for the renewable energy trading scheme effect. Consistent with the baseline results, the estimated effect remains positive and significant for the 2045-or-earlier subgroup, implying an increase of about 77.4% (Table S9).

**Table 1:** NZT legislation status, target year, and interim commitments by country

Country	NZT legislation status	NZT status year	NZT target year	Interim target percentage reduction^a^	Interim target (by 2030)
Finland	In law	2022	2035	60	Emission reduction target
Austria	In law	2021	2040	55	Emission reduction target
Germany	In law	2021	2045	65	Emission reduction target
United Kingdom	In law	2020	2050	68	Emission reduction target
Greece	In law	2022	2050	55	Emission reduction target
Norway	In law	2020	2050	55	Emission reduction target
Ireland	In law	2021	2050	51	Emission reduction target
New Zealand	In law	2019	2050	50	Emission reduction target
Japan	In law	2021	2050	46	Emission reduction target
Australia	In law	2022	2050	43	Emission reduction target
Canada	In law	2021	2050	40	Emission reduction target
France	In law	2019	2050	40	Emission reduction target
Portugal	In law	2021	2050	40	Emission reduction target
Spain	In law	2021	2050	23	Emission reduction target
Poland	Not in law (in policy document)	2019	2030^b^	–	–
United States	Not in law (in policy document)	2021	2050	52	Emission reduction target
Italy	Not in law (in policy document)	2021	2050	43.7	Emission reduction target
Türkiye	Not in law (in policy document)	2021	2053	41	Reduction vs. Business-as-Usual
China	Not in law (in policy document)	2021	2060	65	Emission intensity reduction target
Russia	Not in law (in policy document)	2021	2060	30	Emission reduction target
India	Not in law (in policy document)	2022	2070	45	Emission intensity reduction target
Denmark	Not in law (declaration/pledge)	2020	2045	70	Emission reduction target
Indonesia	Not in law (declaration/pledge)	2021	2050	29	Reduction vs. Business-as-Usual
Israel	Not in law (proposed/in discussion)	2021	2050	27	Emission reduction target

a Interim target percentage reductions are relative to baseline emission years as defined in each country’s legislation or policy document, except where noted. China and India targets refer to emission intensity reductions; Türkiye and Indonesia targets refer to reductions vs. business-*as*-usual.

b Poland’s 2030 NZT target has no interim reduction figure reported.

**Table 2:** The effect of NZT legislation on corporate low-carbon energy investments

Dependent variable^a^	CAPEX^e^ (ln)					
	NZT target=<2050			NZT target =<2045		
	(1)	(2)	(3)	(4)	(5)	(6)
NZTStatus1^b^ × LowCarbon^c^	0.290	0.253	0.061	–	–	–
	(0.48)^d^	(0.41)	(0.11)	–	–	–
NZTStatus2^b^ × LowCarbon	–	–	–	0.668∗∗∗	0.494∗∗∗	0.706∗∗∗
	–	–	–	(5.67)	(4.05)	(6.92)
Observations	5,956	5,956	5,956	5,956	5,956	5,956
Adj. R-squared	0.204	0.221	0.310	0.205	0.221	0.311
Controls	Yes	Yes	Yes	Yes	Yes	Yes
Country FE^f^	Yes	Yes	Yes	Yes	Yes	Yes
Year FE	No	Yes	Yes	No	Yes	Yes
Energy type FE	No	No	Yes	No	No	Yes

a The dependent variable is in natural logarithms.

b NZTStatus1 (NZTStatus2) equals one if the national NZT law has been passed by year t in country c, and the NZT target is set for 2050 (2045) or earlier. NZTStatus1 captures the average association between all statutory NZT targets of 2050 or earlier and low-carbon investment, whereas NZTStatus2 isolates the subset of countries with the most ambitious statutory targets (2045 or earlier). The two indicators are estimated separately and should not be interpreted as mutually exclusive treatment categories.

c LowCarbon equals one if the asset involves solar, wind, hydro, geothermal, battery, or storage technologies.

d Standard errors (SEs) in parentheses are clustered at the country level. ∗∗∗, ∗∗ and ∗ denote significance at the 1%, 5%, and 10% levels respectively.

e CAPEX: capital expenditure.

f FE: fixed effect.

We also investigate whether interim decarbonization targets affect investment behavior. Since most countries in our sample adopted a common interim target year of 2030, we focus on variation in the stringency of interim emission reduction commitments. Ambitious interim targets significantly amplify the positive effect of legally binding NZT laws targeting 2045 or earlier on low-carbon investment (Table S8). In contrast, stringent interim targets alone have no statistically significant effect in the absence of an ambitious NZT legal framework. This suggests that investors respond most strongly when credible long-term net-zero commitments are accompanied by demanding near-term decarbonization milestones.

To address potential endogeneity concerns, we regress the dynamic effects model in Equation 2 (see STAR Methods) to test the pre-existing trends. The estimated coefficient for the pre-legislation indicator is not statistically significant at any conventional level (Tables S10 and S11). After the enactment of ambitious NZT legislation, the post-treatment coefficients become positive and statistically significant. Although the Post1 coefficient is larger than the subsequent Post2+ coefficient, the positive effect persists beyond the first post-legislation year, suggesting that the observed investment response is not limited to a short-term adjustment immediately following enactment. This indicates that the observed positive NZT legislation effects on low-carbon energy investments by public firms are persistent over years and unlikely to be influenced by other climate-related regulations. To reinforce the main results, we conduct various robustness tests with regard to alternative model specifications, variable definitions, and across different subsamples and time periods (Tables S12–S19). The baseline conclusion survived all these sensitivity analyses. We further assess whether the results are driven by any individual treatment country using a leave-one-country-out robustness analysis (Table S22). The subgroup of countries with statutory NZT targets of 2045 or earlier comprises three-treated countries (Germany, Finland, and Austria). We therefore re-estimate the baseline specification after excluding each of these countries one at a time. In every case, the estimated coefficient on NZTStatus2 × LowCarbon remains positive and statistically significant, indicating that the observed relationship is not driven by any single treatment country. Finally, we control for country-level renewable electricity generation to account for differences in the maturity of national electricity systems and the existing level of renewable energy deployment. As reported in Table S23, including this additional control has virtually no effect on the estimated coefficient of NZTStatus2 × LowCarbon, which remains economically and statistically significant. These results confirm that our main findings are robust and are not driven by cross-country differences in the maturity of renewable electricity systems.

### The reinforcing role of NZT disclosure

We further investigate the mechanism through which the national NZT legislation may encourage public firms to allocate more capital to low-carbon energy assets. One potential interpretation is that national plans create an enabling environment for corporate action, setting the overall direction and providing incentives for companies to improve sustainability (e.g., reducing carbon emissions, making low-carbon innovation).^18^

We first examine the role of corporate NZT score measured by climate-related disclosure based upon the variant of the baseline model in Equation 3 (see STAR Methods). The overall firm-level NZT disclosure score we construct comprises four dimensions: targets and planning, finance, policy, and governance. The NZT laws with targeting year no later than 2045 significantly improve the overall NZT disclosure score of public firms, particularly enhancing their disclosure related to NZT targets and planning as well as environmental finance (Figure 2B), while such legislation effects become insignificant for countries targeting 2050 (Figure 2A). More comprehensive firm-level NZT disclosure significantly boosts capital investment in low-carbon power generation assets after the enactment of ambitious NZT laws targeting 2045 or earlier (Figure 2D), however, the effect of the corporate NZT score becomes negligible for countries targeting 2050 (Figure 2C). Specifically, we find that a one standard deviation increase in the overall corporate NZT disclosure score is associated with a 26% increase in capital flows into low-carbon power generation assets (Table S20). Among the four dimensions, the positive effect is more salient regarding environmental finance disclosure.

**Figure 2:**
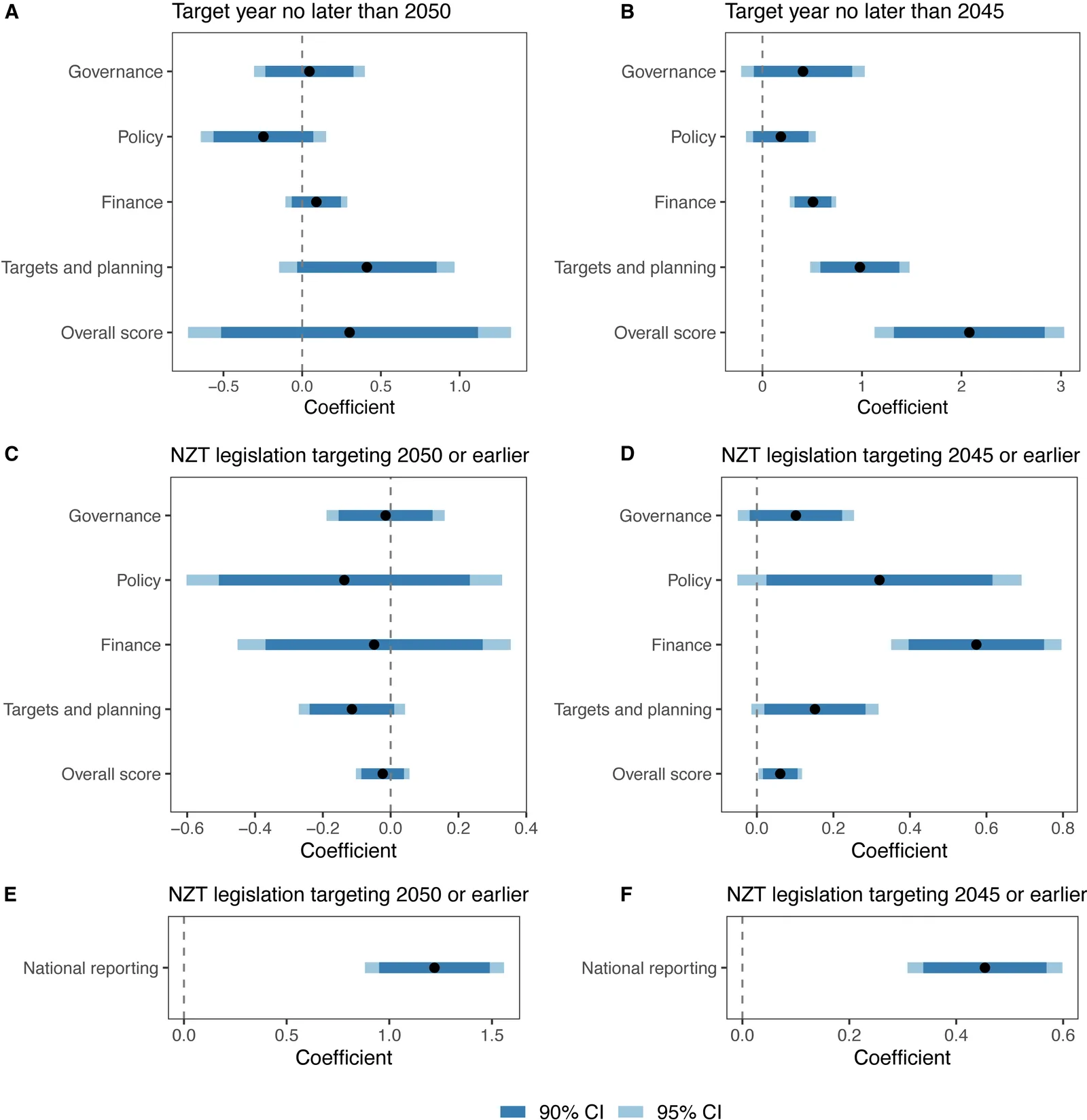
The mechanisms of NZT law-driven low-carbon investments (A) Effects of national NZT laws targeting no later than 2050 (NZTStatus1) on overall corporate NZT score, targets and planning score, finance score, policy score, and governance score. (B) Effects of national NZT laws targeting no later than 2045 (NZTStatus2) on overall corporate NZT score, targets and planning score, finance score, policy score, and governance score. (C) Effects of national NZT legislation aiming for net-zero emissions no later than 2050 on capital investment in low-carbon power assets, moderated by overall corporate NZT score and scores of four dimensions. (D) Effects of national NZT legislation aiming for net-zero emissions no later than 2045 on capital investment in low-carbon power assets, moderated by overall corporate NZT score and scores of four dimensions. (E) Effects of national NZT legislation aiming for net-zero emissions no later than 2050 on capital investment in low-carbon power assets, moderated by national reporting. (F) Effects of national NZT legislation aiming for net-zero emissions no later than 2045 on capital investment in low-carbon power assets, moderated by national reporting. The regression analysis includes 24 countries with 5,956 observations for the period from 2014 to 2023. Points represent estimated regression coefficients. Dark blue error bars denote 90% confidence intervals, and light blue error bars denote 95% confidence intervals.

We then examine whether the frequency of NZT reporting at the national level positively influences how legislation affects public firms' capital allocation to low-carbon technologies (Table S21). Compared with reporting annually, reporting at least twice per year can increase corporate low-carbon energy investments by 58% in countries with an ambitious legally binding NZT target of 2045 or earlier (Figure 2F). In addition, although the enactment of law to achieve net zero by 2050 alone has minimal impact on capital expenditure, pairing it with an NZT reporting mechanism that requires reporting at least twice per year can more than triple capital flows to low-carbon energy assets compared with combining with an annual NZT reporting (Figure 2E).

In a nutshell, we find that the commitment of ambitious legally binding NZT targets boosts low-carbon capital investments through enhanced climate-related disclosure at both firm and country levels.

## Discussion

Our research demonstrates that ambitious legally binding national NZT targets are associated with higher low-carbon energy investment by public firms in recent years. The positive legislation effects are reinforced by transparent and credible firm-level sustainability disclosures and frequent country-level progress reporting (Figure 3). Although countries with legislated NZT targets tend to have higher average GDP per capita, our findings persist after controlling for economic development and investment capacity. This suggests that legislative ambition itself provides an additional signal that influences private capital allocation beyond the advantages associated with higher national income levels. Conversely, in our sample, we do not detect a comparable average investment response to later-dated targets (2050 or later) or to targets articulated outside statute. This null finding should not be interpreted as evidence that such policies never matter; effects may be heterogeneous, take longer to materialize, or operate through other channels beyond the investments observed here.

**Figure 3:**
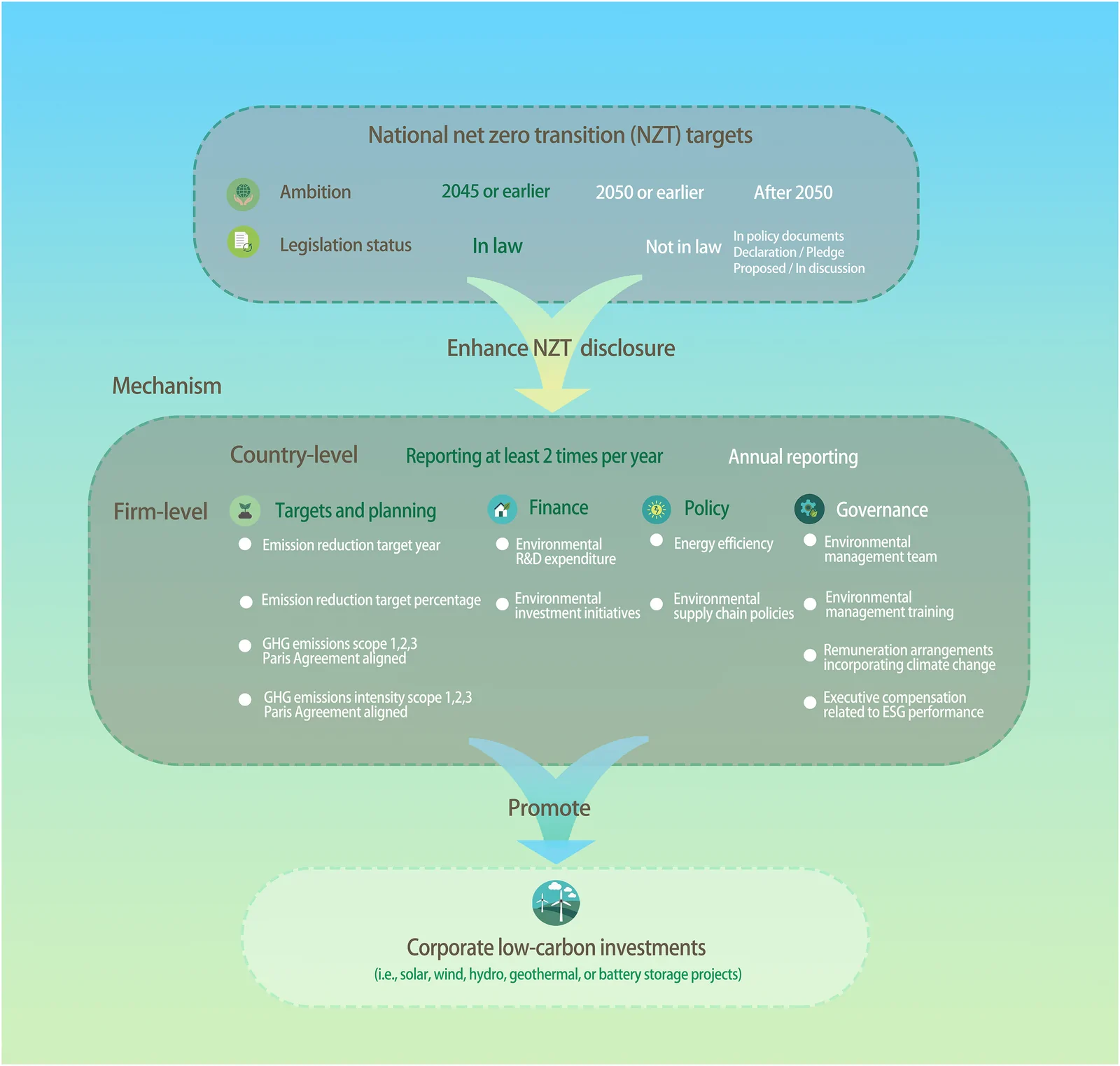
The conceptual framework of national NZT legislation promoting corporate low-carbon investments through enhanced NZT disclosure National NZT targets with ambitious dates in 2045 or earlier and meantime enshrined in law are associated with higher corporate capital flows to solar, wind, and other types of low-carbon energy projects. One interpretation is that according to legitimacy theory, NZT legislation makes countries report progress at least twice per year and pushes firms to disclose more information on emission targets and planning, financial support, and policy implementation, and drive alignment behaviors such as increasing low-carbon investments.

Importantly, these findings are not simply driven by differences in economic development across countries. Although countries with legislated NZT targets tend to have higher average GDP per capita (Table S6), our results remain robust after controlling for GDP per capita, country fixed effects, and firm-level characteristics. This suggests that legislative ambition provides an additional signal that influences private capital allocation beyond the greater investment capacity typically associated with higher national income levels.

Given the fact that large economies and carbon emitters, such as the United States, China, and India, have not committed to achieving net zero by 2050 and their NZT targets have not been enshrined in law and instead remain policy commitments, investments in clean energy may still fall short of the level needed to decarbonize the world’s energy system. More importantly, our regression analysis exclusively focuses on publicly listed firms that tend to face stronger climate pressures from investors and regulators. Yet, previous literature has shown that public firms may sell pollutive plants in response to environmental pressures, and private firms that face weaker environmental pressures and are less in the limelight became the buyer.^19^^,^^20^ This is consistent with our finding that public firms were becoming cleaner over time compared to private firms. This observation highlights an important distinction between capital-market responses and real-economy decarbonization. While statutory NZT legislation may encourage publicly listed firms to increase low-carbon investment and reduce exposure to high-carbon assets, the associated emissions reductions may be limited if carbon-intensive assets are simply transferred to private owners rather than retired, upgraded, or operated under equivalent climate constraints. Effective implementation of NZT strategies therefore requires monitoring ownership transfers and emissions outcomes in both public and private markets.

On the brighter side, enormous advances in the cost-competitiveness of renewables over the past decades have accelerated the new deployment of low-carbon assets and decarbonized the energy mix.^21^^,^^22^ This trend is expected to continue over the following five years (Figure 1); Despite the global reallocation of fossil fuel assets, emerging low-carbon technologies and policy interventions would continue to boost growth in renewable power^23^ and storage capacity,^24^ as well as the use of green electricity globally.

These study findings provide key policy implications at both national and international levels. First of all, legislative ambition matters. Policymakers seeking to mobilize private capital toward decarbonization must move beyond broad 2050 declarations and set intermediate or earlier target dates, ideally enshrined in law. Such legislation can send credible signals to capital markets, reduce regulatory uncertainty, and raise the reputational and financial stakes for non-compliance. In addition, firm-level climate disclosure frameworks should be strengthened and standardized, particularly in jurisdictions that have adopted or plan to adopt NZT legislation. Transparency around targets, policy implementation, and capital alignment enables investors and regulators to assess progress and enforce accountability. Furthermore, the results underscore the importance of aligning NZT legislation with broader institutional and financial ecosystem reforms to ensure that statutory climate targets translate into effective corporate action. Lastly, the research offers guidance for capital market participants and regulators. Lenders and institutional investors should integrate both the national NZT legal status and firm-level disclosure scores into their risk and credit assessments. Firms operating in jurisdictions with ambitious legally binding NZT targets and demonstrating strong sustainability are likely to experience lower transition risk and improved access to capital. For regulators, the capital reallocation in high- and low-carbon energy assets due to different transition risk exposures of public firms and private firms could hinder the accomplishment of NDCs, so the divestment from dirty assets among public firms should be monitored, and the earlier retirement or upgrade of fossil fuel assets^25^ are the more effective solution for reducing losses of stranded assets^26^ and establishing a sustainable energy system.

### Limitations of the study

Despite the robustness of empirical evidence, our study faces certain limitations and calls for further related research. First, the regression models rely on estimated asset-level capital expenditures constructed from technology-, region-, and period-specific unit cost assumptions. Although the results are robust across several alternative specifications and investment measures, some measurement error in the timing and allocation of investment is unavoidable. Second, although the sample for analysis encompasses publicly listed firms across 24 countries, it may not fully capture the global political dynamics that influence the implementation and compliance with climate legislation. Third, we focus only on power generation assets in the energy sector. Future research could work on (1) establishing more comprehensive asset-level investment databases, (2) exploring how climate legislation reshapes high-carbon and low-carbon asset ownership among both public and private firms worldwide, and (3) extending the research scope to other high-emission industries such as cement, steel, and aviation. Researchers could also build on our findings by examining other mechanisms of the NZT legislation effect beyond enhanced disclosure (i.e., the role of financial intermediaries and public-private partnerships). Finally, our analysis focuses on statutory national NZT legislation because legally binding laws provide a more durable and credible policy signal than executive policy commitments or participation in international agreements. Countries such as the United States illustrate this distinction, as climate commitments changed repeatedly through executive actions without corresponding statutory NZT legislation. Examining how such policy reversals affect corporate investment behavior is an important topic for future research but lies beyond the scope of the present study.

## Resource availability

### Lead contact

Requests for further information and resources should be directed to and will be fulfilled by the lead contact, Min Ruan (min.ruan@reuben.ox.ac.uk).

### Materials availability

This study did not generate new unique reagents.

### Data and code availability

• The important data have been deposited at Zenodo and are publicly available as of the date of publication at https://doi.org/10.5281/zenodo.18220988.
• All data reported in this paper will be shared by the lead contact upon request.
• All original code has been deposited at Zenodo at https://doi.org/10.5281/zenodo.18220988 and is publicly available as of the date of publication.
• Any additional information required to reanalyze the data reported in this paper is available from the lead contact upon request.

## Acknowledgments

Funding for this work was provided by Santander Group through their support for the Transition Finance Centre of Excellence. We also acknowledge the input provided by Santander and would like to particularly thank Steffen Kram, Christopher Vernon, Christopher Mogridge, Charlie Liechti, and Etienne Butruille for their feedback. The views expressed in this paper are solely the responsibility of the authors and do not necessarily reflect the opinions of the acknowledged individuals.

## Author contributions

Conceptualization, X.Y.Z., M.R., and G.S.; methodology, X.Y.Z. and M.R.; writing – original draft, X.Y.Z. and M.R.; writing – review and editing, S.F.; funding acquisition, X.Y.Z. and G.S.; resources, X.Y.Z., M.R., and J.Y.; supervision, S.F. and G.S.

## Declaration of interests

The authors declare no competing interests.

## STAR★Methods

### Key resources table

**Table:** 

REAGENT or RESOURCE	SOURCE	IDENTIFIER
**Deposited data**		

National NZT legislation status data	Database: Net Zero Tracker	https://eciu.net/netzerotracker
Environmental policy stringency (EPS) index	Database: OECD	https://data-explorer.oecd.org/
Renewable energy trading scheme index	Database: OECD	https://data-explorer.oecd.org/
GDP per capita data	Database: WRDS	https://wrds-www.wharton.upenn.edu/
Corporate NZT disclosure and financial characteristics data	Database: Refinitiv Eikon	https://eikon.refinitiv.com/
Corporate capital expenditure on low-carbon and high-carbon energy assets	Database: Rystad Energy	https://www.rystadenergy.com/
Complied data for regression analysis	This paper	https://doi.org/10.5281/zenodo.18220988

**Software and algorithms**		

Stata software	Stata	https://www.stata.com/
R software	R	https://www.r-project.org/
Photoshop software	Adobe	https://www.adobe.com/products/photoshop.html

### Method details

We first construct a baseline model to examine the impact of national NZT legislation on corporate capital allocation and conduct a pre-trend analysis to address potential endogeneity and reverse causality. We then carry out mechanism analysis to investigate the moderating role of NZT disclosure in the effect of national NZT legislation on low-carbon investment by incorporating an interaction term into the regression model. Various robustness tests are run across multiple specifications, time windows, investment proxies, and subsamples.

#### Baseline model

Using country-level announcements of NZT commitment as a quasi-natural experiment, which signals national ambition for climate mitigation, we employ a difference-in-differences (DID) approach on asset-level data^27^ to evaluate corporate energy investments. For energy project *j* for firm *i* headquartered in country *c* at year *t*, the baseline regression model is

(Equation 1) $${CAPEX}_{j,i,c,t}=\alpha_{0}+\beta_{1}{NZTStatus}_{c,t}*\;{LowCarbon}_{j}+\beta_{2}{NZTStatus}_{c,t}+{LowCarbon}_{j}+\sum_{2}^{k}\beta_{i}{Control}_{i,c,t}+\gamma_{c}+\lambda_{t}+\delta_{p}+\varepsilon_{j,i,c,t}$$

where *CAPEX*_*j*,*i*,*c*,*t*_ denotes project capital expenditure in natural logarithms. *NZTStatus*_*c*,*t*_ indicates two dummy variables for different statuses of national NZT legislation. It equals one if the national NZT law has been passed by year *t* in country *c*, and the NZT target year is set at 2050 or earlier (*NZTStatus*1_*c*,*t*_) or 2045 or earlier (*NZTStatus*2_*c*,*t*_). The dummy *LowCarbon*_*j*_ equals one if project *j* involves low-carbon technologies (i.e., solar, wind, hydro, geothermal, or battery storage), and zero otherwise. Correspondingly, *β*_1_ captures the effect of national NZT legislation on corporate capital flow to low-carbon projects. We control for firm-level financial characteristics (e.g., size, profitability, leverage) and country-level characteristics (e.g., the number of other climate change regulations/policies, GDP per capita). We also include country fixed effect *γ*_*c*_, year fixed effect *λ*_*t*_ and energy type fixed effect *δ*_*p*_ to control for unobservable country and technology attributes and to absorb year-specific unobservable. *ε*_*j*,*i*,*c*,*t*_ is an unobserved error term. We consistently estimate the model in Equation 1 using ordinary least squares.

#### Endogeneity test

Reverse causality may arise if increases in low-carbon capital expenditure are driven by other climate-related regulations, which would imply an upward trend in such investment prior to the enactment of NZT laws. To ensure that the enactment of NZT laws can be treated as an exogenous event for most firms in our data sample, we conduct an endogeneity test following the approach^27^^,^^28^ to examine the presence of any pre-existing trends. The estimation equation is

(Equation 2) $${CAPEX}_{j,i,c,t}=\alpha_{0}+\alpha_{1}{Pre}_{c,t}^{-2or-1}+\alpha_{2}{Current}_{c,t}^{0}+\alpha_{3}{Post}_{c,t}^{1}+\alpha_{4}{Post}_{c,t}^{+2}+\sum_{2}^{k}\alpha_{i}{Control}_{i,c,t}+\gamma_{c}+\lambda_{t}+\delta_{p}+\varepsilon_{j,i,c,t}$$

where the dummy ${Pre}_{c,t}^{-2or-1}$ equals one if it is one or two years (*t−1* or *t−2*) before an NZT law is passed (at year *t* in country *c*); the dummy ${Current}_{c,t}^{0}$ equals one if it is the year (*t*) when the NZT law is passed in country *c*; the dummy ${Post}_{c,t}^{1}$ equals one if it is one year after NZT law is passed in country *c*; and the dummy ${Post}_{c,t}^{+2}$ equals one if it is two or more years after an NZT law is passed in country *c*. A significant positive coefficient *α*_1_ would suggest that the increase in capital expenditure preceded the law.

#### Mechanism analysis

We investigate whether national NZT legislation affects corporate low-carbon investments by enhancing NZT-related disclosure and alignment behaviour. Using a subsample of low-carbon projects, we propose a variant of the baseline model below:

(Equation 3) $${CAPEX}_{j,i,c,t}=\alpha_{0}+\beta_{1}{NZTStatus}_{c,t}*\;NZT\_Disclosure+\beta_{2}{NZTStatus}_{c,t}+\beta_{3}NZT\_Disclosure+\sum_{2}^{k}\beta_{i}{Control}_{i,c,t}+\gamma_{c}+\lambda_{t}+\delta_{p}+\varepsilon_{j,i,c,t}$$

where *NZT*_*Disclosure* denotes 1) corporate NZT score indexed by *NZT*_*Disclosure*_*i*,*c*,*t*_ and 2) country-level NZT reporting frequency indexed by *NZT*_*Disclosure*_*c*,*t*_. We construct the overall NZT disclosure score at a firm level, by summing up a set of indicator variables from four key dimensions (i.e., target and planning, finance support, policy implementation, and corporate governance). We first test the firm-level overall NZT score effect and then explore the more detailed effects of disclosure by dimension. The dummy *NZT*_*Disclosure*_*ct*_ equals one if country *c* reports more frequently than annually at year *t*, and zero otherwise. In this model, the coefficient *β*_1_ captures the moderating effect of the NZT disclosure score.

#### Robustness checks

To test the stability of the baseline estimates, we conduct a series of robustness checks. First, we employ an alternative proxy for corporate investment in power generation: the number of power plants a firm invests in annually disaggregated by energy type. Second, to examine the possibility that firms can respond rapidly to NZT legislation, we estimate the models over a two-year window. Third, we only focus on solar and wind power projects and assess whether the effects of NZT legislation persist. Fourth, we isolate the influence of potential macroeconomic confounders including national electricity consumption and renewable electricity generation, and fluctuations in oil prices. Fifth, we leave one country out of the treatment group and check whether the results are driven by any individual treatment country.

### Quantification and statistical analysis

All statistical analyses were conducted using Stata and R. The empirical analysis uses plant-firm-country-year observations from 2014 to 2023. The baseline sample contains 5,956 observations for the principal investment regressions, while the exact sample size for each specification is reported in the corresponding table. The regressions are estimated using ordinary least squares with country, year, and energy-type fixed effects, unless otherwise stated. Standard errors are clustered at the country level in all regression specifications to account for within-country serial correlation. No biological materials, human participants, or animal models were used in this study; consequently, sex and gender are not applicable to the analysis.

For the baseline difference-in-differences analysis, the statistical test is based on the interaction between national net-zero transition legislation status and the LowCarbon indicator. The coefficient on this interaction is the parameter of interest and measures the differential association between national NZT legislation and capital expenditure on low-carbon relative to high-carbon power projects. NZTStatus1 identifies statutory net-zero targets of 2050 or earlier, whereas NZTStatus2 identifies statutory targets of 2045 or earlier. For the pre-trend analysis, event-time indicators are estimated relative to the pre-treatment period; statistical inference focuses on whether the pre-treatment coefficients differ significantly from zero. Additional robustness analyses use alternative investment measures, shorter time windows, technology subsamples, additional macroeconomic controls, and leave-one-country-out specifications. Detailed coefficient estimates, standard errors, observations, fixed effects, and adjusted R-squared values are reported in Tables S7–S23.

The mechanism analyses use interaction terms to examine whether corporate NZT disclosure and national NZT reporting frequency reinforce the association between statutory NZT legislation and low-carbon investment. Corporate NZT disclosure is measured as a composite score constructed from 12 binary indicators across targets and planning, finance, policy implementation, and governance. National NZT reporting frequency is measured by a dummy variable equal to one when a country reports progress toward its net-zero target at least twice per year and zero when it reports annually. Exact sample sizes are reported in the relevant tables (for example, 5,776 observations in Tables S20 and S21 and 5,960 observations in Table S19).

For descriptive statistics, the centre of the distribution is reported using the arithmetic mean, with dispersion reported using the standard deviation; the number of observations is reported for each variable (Table S6). Regression estimates are reported with standard errors in parentheses, and statistical significance is indicated at the 1%, 5%, and 10% levels using ∗∗∗, ∗∗, and ∗, respectively. Figure 2 reports point estimates with both 90% and 95% confidence intervals. Figure 1 presents descriptive asset-level patterns rather than inferential statistical tests. The exact n for each regression corresponds to the number of available plant-firm-country-year observations after applying the relevant sample and variable restrictions and is reported in each table.

## Supplemental information

Document S1. Tables S1–S23 and Method S1
