# Supplementary material for: The impact of statutory net-zero targets on clean energy investment
Source: iScience. 2026 Sep 25;29(10):117647. doi: 10.1016/j.isci.2026.117647 (PMC13635561; doi:10.1016/j.isci.2026.117647)
Supplement: Supplementary file 1 [file mmc1.pdf]

iScience, Volume *29*

Supplemental information

The impact of statutory net-zero targets on clean energy investment

Xiao Yan Zhou, Min Ruan, Jing Yu, Sam Fankhauser, and Gireesh Shrimali

Table S1. The number, energy type, and capital expenditure of power plants

Power plant	Capex (Mean) million USD	Capex (Sum) million USD	N
Solar PV	42	186330	4442
Onshore wind	61	87281	1422
Battery	48	26243	544
Offshore wind	388	135541	349
Gas	378	39332	104
Coal	783	62673	80
Hydro	408	19989	49
Pumped storage	1115	22308	20
Geothermal	95	1518	16
Solar thermal	182	2189	12
Bioenergy	649	5194	8

Note: Rystad Energy contains annual information on plant-level variables that are central to our analysis. More specifically, Rystad Energy contains plant-level information on plant name, ID, size (capacity measured in MW), energy type, location (latitude, longitude), country of location, company owners, country of company headquarter, and share ownership. The Rystad databases offer their estimated capital expenditures (Capex) for renewable power plants, mainly solar PV and wind power plants. Based on their estimates, we filled out the missing values based on the fitted line of plant capacity and Capex by energy type and first production year. When it comes to fossil fuel power plants, we referred to the Capex data from US NREL and conservatively estimated the Capex based on plant capacity for coal power (~$3000 per kW), natural gas or combined cycle (~$1000 per kW), and fuel cells (~$6000 per kW).

Table S2. The estimated capital investment cost ($/kW, 2020 USD) by energy type, continent and year

Technology	Year	Asia	Europe	N. America	S. America	Africa	Oceania
**Coal**	2000	~800–1,200	~1,200–1,800	~1,000–1,500	~1,200–1,500	~1,500+	~1,200–1,500
(Pulverized coal)	2010	~1,000–1,500	~1,800–2,500	~2,000–2,500	~1,500–2,000	~2,000+	~2,000
	2020	~1,000–1,300	~2,500–4,000	~3,000+	~2,000–3,000	~2,500–4,000	~2,500
	2025*	~1,100 (efficient)	– (phasing out)		~2,200	~2,700	
**Natural Gas**	2000	~400–600	~500–800	~500–700	~600–800	~700+	~600–800
(Combined cycle)	2010	~500–800	~800–1,000	~900–1,000	~800–900	~1,000+	~900
	2020	~600–900	~1,000–1,300	~900–1,100	~800–1,000	~1,200	~1,000
	2025*	~650	~1,100	~950	~900	~1,200+	~1,000
**Oil-fired**	2000	~300–500 (diesel)	~400–800	~500–800	~600–900	~600–1,000	~500–800
	2020	~400–700	~800–1,000	~600–900	~800–1,000	~1,000+	~800–1,000
**Solar PV**	2000	>10,000	>10,000	>10,000	>12,000	>15,000	>10,000
(Utility-scale)	2010	~3,000–4,000	~4,000–5,000	~4,500	~4,000+	~5,000+	~4,000+
	2020	600–800	~1,100–1,400	~1,000–1,300	~900–1,100	~1,200+	~1,100
	2025*	500–600	~1,000	~800–1,000	~800	~1,000+	~900
**Solar Thermal**	2000	~8000	~9000	~8000	~9000	–	–
	2010	~7000	~8000	~6000	~9000	–	~8000
	2020	~5000	~7000	~7500	~8000	~6500	~7000
	2025*	~4000	~6000	~6000	~6000	~5000	~6000
**Onshore Wind**	2000	~1,200–1,500	~1,300–1,600	~1,200–1,500	~1,500	~1,800	~1,500
	2010	~1,100–1,300	~1,700–2,000	~1,800	~1,500–1,800	~2,000+	~1,800
	2020	1,050–1,100	~1,600–1,800	~1,300–1,600	~1,300–1,500	~1,600–2,000	~1,500–1,600
	2025*	~1,000	~1,500	~1,400	~1,200	~1,500+	~1,400
**Offshore Wind**	2000	–	~5,000–6,000	–	–	–	–
	2010	–	~5,000–5,500	–	–	–	–
	2020	~2,500–3,000	~3,000–4,500	~3,500	–	–	–
	2025*	~2,000–2,500	~2,500–3,000	~3,000	~3,000	~3,500	~3,000
**Nuclear**	2000	~2,000–3,500	~2,500–4,000	~2,000–3,000	–	–	–
(Large PWR)	2010	~2,500–4,000	~4,000–6,000	~4,000+	–	–	–
	2020	~2,500–5,500	~6,000–10,000	~6,000–8,000	–	–	–
	2025*	~3,000–6,000	~8,000+	~7,000+	–	–	–
**Large Hydro**	2000	~1200	~3000	~2500	~1800	~2500	~3000
**(>50MW)**	2010	~1500	~4000	~3000	~2000	~2500	~3500
	2020	~2200	~5000	~4000	~2200	~3000	~4000
	2025*	~2500	~5500	~4500	~2500	~3200	~4500
**Small Hydro**	2000	~2000	~5000	~4000	~3000	~4000	~6000
**(<50MW)**	2010	~2500	~6000	~5000	~3500	~4500	~7000
	2020	~3000	~7000	~6000	~4000	~5000	~8000
	2025*	~3200	~7500	~6500	~4500	~5500	~8500
**Biomass**	2000	~2500	~3000	~2500	~2000	~3000	~3500
	2010	~2000	~3500	~3000	~2500	~3200	~4000
	2020	~1800	~4000	~3500	~3000	~3500	~4500
	2025*	~2000	~4200	~3700	~3200	~3700	~4800
**Geothermal**	2000	~2500	~4000	~3000	~4000	~5000	~3000

	2010	~3000	~4500	~3500	~3500	~4500	~3500
	2020	~3500	~5000	~4500	~4000	~5000	~4000
	2025*	~3800	~5300	~4800	~4200	~5500	~4200
**Pumped Storage**	2000	~1000	~2000	~1500	~1500	~1800	~2000
	2010	~1200	~2500	~1800	~1800	~2000	~2200
	2020	~1500	~3000	~2500	~2200	~2600	~4000
	2025*	~1700	~3300	~2700	~2500	~3000	~4500
**Hydrogen**	2000	~4000	~4500	~4000	~5000	~5000	~4500
**Electrolyzer**	2010	~3000	~3500	~3000	~4000	~4000	~3500
	2020	~900	~1200	~1000	~1500	~1500	~1200
	2025*	~500	~700	~600	~700	~800	~700

Notes: Figures marked with * are projections based on current trends or targets. “–” indicates scarce data or no notable projects. Sources: IEA World Energy Outlook data and IRENA (2022), Renewable Technology Innovation Indicators: Mapping progress in costs, patents and standards, International Renewable Energy Agency, Abu Dhabi. ISBN: 978-92-9260-424-0. 2025

Table S3. National net zero transition (NZT) legislation status data

	National NZT status	NO. of countries	NZT target year=2050	NZT target year<2050	NZT target year>2050
Original NZT legislation data	In law	34	22	10	4
	Not in law	146	98	38	10
	In policy documents	82	44	32	6
	Proposed / in discussion	44	42	1	1
	Declaration / pledge	20	12	5	3
After combination of national risk factors and company data	In law	14	11	3	
	Not in law	10	4	2	7
	In policy documents	7	2	1	4
	Proposed / in discussion	1	1		
	Declaration / pledge	2	1	1	3

Notes: We chose the legislated target year 2050 as a cutoff point because many countries are aiming for 2050 for net-zero emissions to align with scientific recommendations for limiting global warming to 1.5°C above pre-industrial levels. However, some countries are more ambitious and are setting a NZT target by 2045, which involves significant and rapid emission reduction according to some climate models17. Thus, we also use the legislated target year of 2045 or earlier to identify the indicator variable of legislation status.

Table S4. Corporate NZT score variables

Variables	Variable description
NZT finance	Measure of a borrower's net zero transition financial support which include environmental R&D expenditure, environmental expenditure investments, and environmental investments initiatives.
Env R&D expenditure	Indicator that equals to one if the borrower has disclosed environmental R&D expenditure.
Env expenditure	Indicator that equals to one if the borrower has disclosed environmental expenditure investments.
NZT target and planning	Measure of a borrower's net zero transition target and planning which include emission target year, percentage of reduction target, long-term net zero target, emission and emission intensity alignment with Paris agreement.
Target year	Indicator that equals to one if the borrower disclosed emission reduction target year.
Target %	Indicator that equals to one if the borrower disclosed the percentage of emission reduction target.
Paris agreement aligned (emission scope 1,2 3)	Indicator that equals to one if the borrower's emissions (scope 1,2,3) reduction target aligned with Paris Agreement.
Paris agreement aligned (emission intensity)	Indicator that equals to one if the borrower's emissions intensity reduction target aligned with Paris Agreement.
NZT policies	Measure of a borrower's policy support on net zero transition
Policy energy efficiency	Indicator that equals to one if the borrower has energy efficiency policy
Policy Env supply chain	Indicator that equals to one if the borrower has environmental policy in supply chain
NZT governance	Measure of borrower's governance support on net zero transition
Env management team	Indicator that equals to one if the borrower has environmental management team.
Env management training	Indicator that equals to one if the borrower has environmental management training.
Remuneration climate change	Indicator that equals to one if the borrower's remuneration arrangement incorporate climate change.
Executive compensation	Indicator that equals to one if the borrower's executive compensation incorporate climate change.
NZT overall score	The overall score of a borrower's overall net zero transition disclosure

Notes: We chose the legislated target year 2050 as a cutoff point because many countries are aiming for 2050 for net-zero emissions to align with scientific recommendations for limiting global warming to 1.5°C above pre-industrial levels. However, some countries are more ambitious and are setting a NZT target by 2045, which involves significant and rapid emission reduction according to some climate models17. Thus, we also use the legislated target year of 2045 or earlier to identify the indicator variable of legislation status.

Table S5. The description of important variables

Variables	Variable description	Data sources
NZTStatus	It comprises two dummy variables that represent the different statuses of the national NZT legislation. NZTStatus1 equals one if the national NZT law has been passed by year t in country c, and the NZT target is set for 2050 or earlier. NZTStatus2 equals one if the national NZT law has been passed by year t in country c, and the NZT target is set for 2045 or earlier.	The Net Zero Tracker
Capital expenditure	The capital expenditure on power plants	Rystad Energy
Corporate net zero transition score	Corporate net zero transition measures include a variety of strategies and actions that companies undertake to reduce their greenhouse gas (GHG) emissions to net zero, covering four key NZT aspects: targets and strategies, financial actions, emission reduction policies and governances (Table IA6).	Eikon
National NZT reporting	It is dummy variable. It equals to 1 if the country report net zero transition progress at least twice per year and equals to zero if it reports NZT progress annually.	The Net Zero Tracker
Controls		
Size	Logarithm of the market value of a company	OECD data
Profit	Gross profit margin	Eikon
Leverage	Ratio of total debt over total assets	Eikon
EPS	The environmental policy stringency index downloaded form OCED database	Eikon
Log (GDP Per Capita)	The logarithm of GDP per Capita	WRDS

Table S6. Summary statistics of important variables

Variable	All sample			National NZT in law			National NZT not in law		
	Mean	St.D	Obs	Mean	St.D	Obs	Mean	St.D	Obs
	(1)	(2)	(3)	(4)	(5)	(6)	(7)	(8)	(9)
ln(CAPEX)	3.02	1.76	5956	3.131	1.576	2103	2.954	1.843	3853
Firm NZT overall score	4.96	3.78	5956	6.529	3.980	2103	4.110	3.374	3853
Firm NZT target	0.93	1.34	5956	1.522	1.547	2103	0.604	1.080	3853
Firm NZT finance	0.45	0.62	5956	0.636	0.682	2103	0.344	0.560	3853
Firm NZT policy	1.20	0.86	5956	1.417	0.844	2103	1.079	0.838	3853
Firm NZT Governance	2.39	1.69	5956	2.954	1.661	2103	2.083	1.632	3853
Low carbon plants	0.97	0.17	5956	0.978	0.146	2103	0.965	0.183	3853
EPS index	3.16	0.70	5956	3.548	0.895	2103	2.941	0.445	3853
GDP per capita	31415	23211	5956	43471	15999	2103	24834	23886	3853
log(MV)	9.13	1.67	5956	9.198	1.876	2103	9.094	1.550	3853
Profit margin	0.04	0.69	5956	0.032	0.738	2103	0.038	0.667	3853
Leverage	2.20	1.99	5956	1.637	1.318	2103	2.506	2.220	3853

Note: Plant-level data are taken from the Rystad Energy data match for the years 2014–2023. "All sample" refers to all plants in the sample; "National NZT in law" is the subset of plants that belong to firms incorporated in a country with NZT in law; "National NZT not in law" is the subset of plants that belong to firms incorporated in a country with other NZT status (policy documents, proposed in discussion, and Declaration/pledge). Leverage is the rate of total debts to total assets. GDP per capita is expressed in thousands of dollars.

Table S7. National NZT legislation and capital expenditure on power plants

	CAPEX to power plants (ln)					
	NZT target=<2050			NZT target =<2045		
	(1)	(2)	(3)	(4)	(5)	(6)
NZTStatus1*LowCarbon	0.290	0.253	0.061			
	(0.48)	(0.41)	(0.11)			
**NZTStatus2*Low Carbon**				**0.668*** **	**0.494*** **	**0.706*** **
				**(5.67)**	**(4.05)**	**(6.92)**
NZT Status1	-0.367	-0.368	-0.230			
	(-0.66)	(-0.62)	(-0.44)			
Low carbon	-2.773***	-2.804***		-2.743***	-2.778***	
	(-12.20)	(-12.35)		(-13.09)	(-13.16)	
EPS	-0.218	0.168	0.120	-0.274	0.143	0.084
	(-0.75)	(1.02)	(0.70)	(-1.08)	(0.96)	(0.54)
GDP per capita	0.000**	0.000*	0.000*	0.000**	0.000*	0.000*
	(2.37)	(2.02)	(2.04)	(2.18)	(1.91)	(1.88)
MV	0.155**	0.158**	0.096	0.155**	0.160**	0.098
	(2.48)	(2.45)	(1.58)	(2.48)	(2.49)	(1.62)
Profit margin	0.111**	0.103**	0.143***	0.108**	0.100**	0.139***
	(2.59)	(2.36)	(3.13)	(2.55)	(2.28)	(3.00)
Leverage	-0.051*	-0.051**	-0.046**	-0.049*	-0.049**	-0.044**
	(-1.98)	(-2.13)	(-2.47)	(-1.91)	(-2.08)	(-2.30)
Country FE	YES	YES	YES	YES	YES	YES
Year FE	NO	YES	YES	NO	YES	YES
Energy type FE	NO	NO	YES	NO	NO	YES
Observation	5956	5956	5956	5956	5956	5956
adj. R-sq	0.204	0.221	0.310	0.205	0.221	0.311

Note: This table reports the results relating corporate power plant investments to the enactment of NZT Laws. Specifically, we estimate the OLS model of the natural logarithm of capital expenditure on an indicator variable of NZTStatus*LowCarbon. NZTStatus comprises two dummy variables that represent the different statuses of the national NZT legislation. NZTStatus1 in Columns (1) to (3) equals one if the national NZT law has been passed by year t in country c, and the NZT target is set for 2050 or earlier. NZTStatus2 in Columns (4) to (6) equals one if the national NZT law has been passed by year t in country c, and the NZT target is set for 2045 or earlier. Low carbon is a dummy variable and equals one if the project involves solar, wind, hydro, geothermal, battery, or storage technologies. EPS is the environmental policy stringency index downloaded from the OECD database. Plant-level data are sourced from Rystad. We also control for GDP per capita and firm-level characteristics, including market value, profit margin, and leverage. All regressions are estimated with year, country, and energy type fixed effects (FE), and the standard errors (SEs) are clustered at the country level. Data is for the period 2014 to 2023. ***, ** and * denote significance at the 1%, 5%, and 10% levels respectively.

Table S8. Interim national NZT effect on capital flow to low carbon power plants

	CAPEX to power plants (ln)	
	NZT target=<2045	NZT target all
	(1)	(2)
NZTStatus2* Interim_high*LowCarbon	0.706***	
	(6.92)	
Interim_high*LowCarbon		0.088
		(0.18)
Renewable energy trading scheme	0.084	0.084
	(0.54)	(0.55)
GDP per capita	0.000*	0.000*
	(1.88)	(1.88)
MV	0.098	0.098
	(1.62)	(1.64)
Profit margin	0.139***	0.140***
	(3.00)	(3.02)
Leverage	-0.044**	-0.045**
	(-2.30)	(-2.33)
Country fixed effect	YES	YES
Year fixed effect	YES	YES
Energy type fixed effect	YES	YES
Observation	5956	5956
adj. R-sq	0.311	0.310

Note: This table reports the results relating corporate power plant investments to the enactment of NZT Laws. In column (1), we estimate the OLS model of the natural logarithm of capital expenditure on an indicator variable of NZTStatus2* Interim_high*LowCarbon. NZTStatus2 equals one if the national NZT law has been passed by year t in country c, and the NZT target is set for 2045 or earlier. Interim_high equals one if the national interim NZT is set to 2030 and the reduction target exceeds the median of 46%. Low carbon is a dummy variable and equals one if the project involves solar, wind, hydro, geothermal, battery, or storage technologies. In column (2), we estimate the OLS model of the natural logarithm of capital expenditure on the interim effect (Interim_high*LowCarbon) regardless of NZT target legislation. Renewable energy trading scheme index downloaded from the OECD database. Plant-level data are sourced from Rystad. We also control for GDP per capita and firm-level characteristics, including market value, profit margin, and leverage. All regressions are estimated with year, country, and energy type fixed effects (FE), and the standard errors (SEs) are clustered at the country level. Data is for the period 2014 to 2023. ***, ** and * denote significance at the 1%, 5%, and 10% levels respectively.

Table S9. National NZT legislation and capital expenditure on low carbon power plants

	CAPEX to low carbon (ln)					
	National NZT target=<2050			National NZT target =<2045		
	(1)	(2)	(3)	(4)	(5)	(6)
NZTStatus1	-0.153	-0.094	-0.079			
	(-0.84)	(-0.62)	(-0.48)			
**NZTStatus2**				**0.656*** **	**0.573*** **	**0.574*** **
				**(5.77)**	**(6.41)**	**(4.77)**
Renewable energy trading scheme	0.229***	0.273***	0.294***	0.235***	0.291***	0.316***
	(5.00)	(4.58)	(4.01)	(4.88)	(5.12)	(4.38)
GDP per capita	0.000*	0.000	0.000*	0.000	0.000	0.000*
	(1.88)	(1.56)	(1.94)	(1.65)	(1.50)	(1.86)
MV	0.147**	0.153**	0.087**	0.146**	0.154**	0.079*
	(2.38)	(2.27)	(2.10)	(2.40)	(2.29)	(1.75)
Profit margin	0.117**	0.106**	0.042	0.113**	0.104**	0.035
	(2.78)	(2.49)	(0.83)	(2.76)	(2.38)	(0.66)
Leverage	-0.063**	-0.060**	-0.008	-0.062**	-0.059**	-0.004
	(-2.29)	(-2.35)	(-0.18)	(-2.25)	(-2.31)	(-0.10)
Country FE	YES	YES	YES	YES	YES	YES
Year FE	NO	YES	YES	NO	YES	YES
Energy type FE	NO	NO	YES	NO	NO	YES
Observation	5776	5776	5741	5776	5776	5741
adj. R-sq	0.135	0.155	0.308	0.135	0.155	0.309

Note: This table reports the results relating corporate investments in low carbon power plants to the enactment of NZT Laws over a 2-year window. Specifically, we estimate the OLS model of the logarithm of capital expenditure on an indicator variable of NZTStatus. It comprises two dummy variables that represent the different statuses of the national NZT legislation. NZTStatus1 in Columns (1) to (3) equals one if the national NZT law has been passed by year t in country c, and the NZT target is set for 2050 or earlier. NZTStatus2 in Columns (4) to (6) equals one if the national NZT law has been passed by year t in country c, and the NZT target is set for 2045 or earlier. LowCarbon is a dummy variable and equals one if the project involves solar, wind, hydro, geothermal, battery, or storage technologies. Renewable energy trading scheme index downloaded from the OECD database. Plant-level data are sourced from Rystad. We also control for GDP per capita and firm-level characteristics, including market value, profit margin, and leverage. All regressions are estimated with year, country, and energy type fixed effects, and the standard errors are clustered at the country level. Data is for the period 2014 to 2023. ***, ** and * denote significance at the 1%, 5%, and 10% levels respectively.

Table S10. National NZT legislation and capital expenditure dynamics

	CAPEX to low carbon (ln)	
	National net zero target=<2050	National net zero target =<2045
	(1)	(2)
**Pre^{-2or-1}**	-0.020	0.442
	(-0.16)	(1.31)
**Current^{0}**	-0.139	0.303**
	(-1.17)	(2.43)
**Post^{1}**	-0.297	1.722***
	(-0.68)	(6.75)
**Post^{2+}**	-0.221	0.795***
	(-1.02)	(6.82)
EPS	0.135	0.117
	(0.77)	(0.69)
GDP per capita	0.000*	0.000*
	(1.86)	(1.86)
MV	0.096	0.097
	(1.60)	(1.59)
Profit margin	0.151***	0.148***
	(3.32)	(3.16)
Leverage	-0.051**	-0.049**
	(-2.70)	(-2.59)
Country FE	YES	YES
Year FE	YES	YES
Energy type FE	YES	YES
Observations	5776	5776
adj. R-squared	0.251	0.252

Notes: This table reports the results relating corporate investments in low carbon projects to the passing of national net zero transition laws. Specifically, we estimate an OLS model of the natural logarithm of capital expenditure on four indicators: Pre^{-2or-1} , Current^{0}, Post^{1} , and Post^{2+}. In column (1), the national NZT law has been passed and the NZT target is to be achieved by 2050 (including 2050). In Column (2), the national NZT law has been passed, and the NZT target is to be achieved by 2045 (including 2045). LowCarbon is a dummy variable and equals one if solar, wind, hydro, geothermal, battery, or storage projects. EPS is the Environmental Policy Stringency Index, downloaded from the OECD database. We also control for GDP per capita and firm level characteristics, including market value, profit margin and leverage. All regressions are estimated with year, country and energy type fixed effects and the standard errors are clustered at the country level. Data is for the period 2014 to 2023. ***, ** and * denote significance at the 1%, 5% and 10% levels respectively.

Table S11. Extended pre-trend test of national NZT legislation and capital expenditure on low-carbon power plants

	CAPEX to low carbon (ln)		
	National net zero target=<2045		
	(1)	(2)	(3)
**Pre^{-2or-1}**	0.267	0.313	0.329
	(0.81)	(0.97)	(0.99)
**Pre^{-3or-4}**		0.134	0.151
		(1.40)	(1.36)
**Pre^{-5or-6}**			0.067
			(0.46)
EPS	0.116	0.119	0.120
	(0.70)	(0.71)	(0.71)
GDP per capita	0.000*	0.000*	0.000*
	(1.85)	(1.85)	(1.84)
MV	0.097	0.097	0.097
	(1.61)	(1.61)	(1.61)
Profit margin	0.148***	0.148***	0.148***
	(3.17)	(3.17)	(3.17)
Leverage	-0.050**	-0.050**	-0.050**
	(-2.69)	(-2.68)	(-2.68)
Country fixed effect	YES	YES	YES
Year fixed effect	YES	YES	YES
Energy type fixed effect	YES	YES	YES
Observation	5776	5776	5776
adj. R-sq	0.250	0.250	0.250

Notes: This table extends the pre-trend analysis reported in Table S9 by including indicators for three-to-four years before legislation (Pre-3or-4) and five-to-six years before legislation (Pre-5or-6). The dependent variable is the natural logarithm of capital expenditure on low-carbon power plants. All regressions include country, year, and energy-type fixed effects, and standard errors are clustered at the country level.

Table S12. National NZT legislation and capital expenditure on power plants: alternative measure

	NO. of power plants	
	National net zero target=<2050	National net zero target =<2045
	(1)	(2)
NZTStatus1*LowCarbon	-4.090	
	(-1.06)	
**NZTStatus2*Low Carbon**		9.699**
		(2.67)
NZTStatus1	10.854	
	(1.61)	
EPS	-16.268	-14.781
	(-1.31)	(-1.32)
GDP per capita	0.000*	0.000
	(1.85)	(1.59)
MV	4.235**	4.103**
	(2.22)	(2.22)
Profit margin	-2.660*	-2.548
	(-1.75)	(-1.70)
Leverage	1.434**	1.393**
	(2.72)	(2.60)
Country FE	YES	YES
Year FE	YES	YES
Energy type FE	YES	YES
Observation	5956	5956
adj. R-sq	0.423	0.421

Note: This table reports the results relating alternative measures of corporate power plant investments to the enactment of NZT Laws. Specifically, we estimate the OLS model of the number of power plants by energy type that a firm invests in during a year on an indicator variable of NZTStatus*LowCarbon. NZTStatus comprises two dummy variables that represent the different statuses of the national NZT legislation. NZTStatus1 in Column (1) equals one if the national NZT law has been passed by year t in country c, and the NZT target is set for 2050 or earlier. NZTStatus2 in Column (2) equals one if the national NZT law has been passed by year t in country c, and the NZT target is set for 2045 or earlier. LowCarbon is a dummy variable and equals one if the project involves solar, wind, hydro, geothermal, battery, or storage technologies. EPS is the Environmental Policy Stringency Index, downloaded from the OECD database. Plant-level data are sourced from Rystad. We also control for GDP per capita and firm-level characteristics, including market value, profit margin, and leverage. All regressions are estimated with year, country, and energy type fixed effects, and the standard errors are clustered at the country level. Data is for the period 2014 to 2023. ***, ** and * denote significance at the 1%, 5%, and 10% levels respectively.

Table S13. National NZT legislation and corporate expenditure on low carbon power plants: alternative measure

	NO. of low carbon power plants	
	National net zero target=<2050	National net zero target =<2045
	(1)	(2)
NZTStatus1	7.136	
	(1.71)	
**NZTStatus2**		10.047**
		(2.70)
EPS	-17.072	-15.401
	(-1.33)	(-1.33)
GDP per capita	0.000*	0.000*
	(2.00)	(1.75)
MV	4.291**	4.148**
	(2.22)	(2.21)
Profit margin	-2.761*	-2.635*
	(-1.79)	(-1.74)
Leverage	1.487**	1.445**
	(2.76)	(2.62)
Country FE	YES	YES
Year FE	YES	YES
Energy type FE	YES	YES
Observation	5776	5776
adj. R-sq	0.416	0.413

Note: This table reports the results relating alternative measures of capital investments in low carbon power plants to the enactment of NZT Laws. Specifically, we estimate the OLS model of the number of power plants by energy type that a firm invests in during a year on an indicator variable of NZTStatus. It comprises two dummy variables that represent the different statuses of the national NZT legislation. NZTStatus1 in Columns (1) equals one if the national NZT law has been passed by year t in country c, and the NZT target is set by 2050. NZTStatus2 in Columns (4) equals one if the national NZT law has been passed by year t in country c, and the NZT target is set by 2045. EPS is the environmental policy stringency index downloaded from the OECD database. Plant-level data are sourced from Rystad. We also control for GDP per capita and firm-level characteristics, including market value, profit margin, and leverage. All regressions are estimated with year, country, and energy type fixed effects, and the standard errors are clustered at the country level. Data is for the period 2014 to 2023. ***, ** and * denote significance at the 1%, 5%, and 10% levels respectively.

Table S14. National NZT legislation and capital expenditure on power plants over a 2-year window.

	CAPEX to low carbon power plants (ln)					
	NZT target=<2050			NZT target =<2045		
	(1)	(2)	(3)	(4)	(5)	(6)
NZTStatus1*LowCarbon	0.323	0.332	0.105			
	(0.46)	(0.47)	(0.16)			
NZTStatus2*LowCarbon				0.714***	0.485***	0.548***
				(4.69)	(3.56)	(3.47)
NZTStatus1	-0.400	-0.446	-0.308			
	(-0.62)	(-0.69)	(-0.53)			
LowCarbon	-2.886***	-2.955***		-2.841***	-2.909***	
	(-7.68)	(-8.16)		(-8.72)	(-9.24)	
EPS	-0.275	0.039	0.124	-0.376**	-0.039	-0.006
	(-1.04)	(0.13)	(0.43)	(-2.47)	(-0.14)	(-0.03)
GDP per capita	0.000**	0.000	0.000	0.000**	0.000	0.000
	(2.29)	(1.43)	(1.47)	(2.18)	(1.37)	(1.35)
MV	0.142*	0.145*	0.084	0.141*	0.146*	0.085
	(2.06)	(2.07)	(1.43)	(2.05)	(2.07)	(1.45)
Profit margin	0.121*	0.107	0.139**	0.118*	0.104	0.135**
	(1.91)	(1.70)	(2.20)	(1.88)	(1.64)	(2.10)
Leverage	-0.045	-0.045	-0.035	-0.045	-0.044	-0.034
	(-1.43)	(-1.50)	(-1.58)	(-1.42)	(-1.49)	(-1.53)
Country FE	YES	YES	YES	YES	YES	YES
Year FE	NO	YES	YES	NO	YES	YES
Energy type FE	NO	NO	YES	NO	NO	YES
Observation	4833	4833	4833	4833	4833	4833
adj. R-sq	0.198	0.212	0.302	0.199	0.212	0.302

Note: This table reports the results relating corporate power plant investments to the enactment of NZT Laws over a 2-year window. Specifically, we estimate the OLS model of the logarithm of capital expenditure on an indicator variable of NZTStatus*LowCarbon. NZTStatus comprises two dummy variables that represent the different statuses of the national NZT legislation. NZTStatus1 in Columns (1) to (3) equals one if the national NZT law has been passed by year t in country c, and the NZT target is set for 2050 or earlier. NZTStatus2 in Columns (4) to (6) equals one if the national NZT law has been passed by year t in country c, and the NZT target is set for 2045 or earlier. LowCarbon is a dummy variable and equals one if the project involves solar, wind, hydro, geothermal, battery, or storage technologies. EPS is the environmental policy stringency index downloaded from the OECD database. Plant-level data are sourced from Rystad. We also control for GDP per capita and firm-level characteristics, including market value, profit margin, and leverage. All regressions are estimated with year, country, and energy type fixed effects, and the standard errors are clustered at the country level. Data is for the period 2014 to 2023. ***, ** and * denote significance at the 1%, 5%, and 10% levels respectively.

Table S15. National NZT legislation and capital expenditure on low carbon power plants over a 2-year window.

	CAPEX to low carbon power plants (ln)					
	NZT target=<2050			NZT target =<2045		
	(1)	(2)	(3)	(4)	(5)	(6)
NZTStatus1	-0.131	-0.172	-0.262			
	(-0.58)	(-0.93)	(-1.39)			
NZTStatus2				0.697***	0.467***	0.527***
				(4.46)	(3.33)	(3.32)
EPS	-0.198	0.110	0.189	-0.346**	0.000	0.027
	(-0.67)	(0.33)	(0.60)	(-2.09)	(0.00)	(0.12)
GDP per capita	0.000**	0.000	0.000	0.000**	0.000	0.000
	(2.29)	(1.43)	(1.48)	(2.14)	(1.36)	(1.34)
MV	0.139*	0.143*	0.080	0.139*	0.145*	0.082
	(2.01)	(2.02)	(1.36)	(2.01)	(2.03)	(1.39)
Profit margin	0.127*	0.113*	0.147**	0.123*	0.110*	0.142**
	(2.01)	(1.81)	(2.36)	(1.96)	(1.73)	(2.23)
Leverage	-0.050	-0.049	-0.042*	-0.050	-0.048	-0.041*
	(-1.55)	(-1.62)	(-1.93)	(-1.54)	(-1.61)	(-1.89)
Country FE	YES	YES	YES	YES	YES	YES
Year FE	NO	YES	YES	NO	YES	YES
Energy type FE	NO	NO	YES	NO	NO	YES
Observation	4717	4717	4717	4717	4717	4717
adj. R-sq	0.143	0.158	0.257	0.144	0.158	0.257

Note: This table reports the results relating corporate investments in low carbon power plants to the enactment of NZT Laws over a 2-year window. Specifically, we estimate the OLS model of the logarithm of capital expenditure on an indicator variable of NZTStatus. It comprises two dummy variables that represent the different statuses of the national NZT legislation. NZTStatus1 in Columns (1) to (3) equals one if the national NZT law has been passed by year t in country c, and the NZT target is set for 2050 or earlier. NZTStatus2 in Columns (4) to (6) equals one if the national NZT law has been passed by year t in country c, and the NZT target is set for 2045 or earlier. LowCarbon is a dummy variable and equals one if the project involves solar, wind, hydro, geothermal, battery, or storage technologies. EPS is the environmental policy stringency index downloaded from the OECD database. Plant-level data are sourced from Rystad. We also control for GDP per capita and firm-level characteristics, including market value, profit margin, and leverage. All regressions are estimated with year, country, and energy type fixed effects, and the standard errors are clustered at the country level. Data is for the period 2014 to 2023. ***, ** and * denote significance at the 1%, 5%, and 10% levels respectively.

Table S16. National NZT legislation on corporate expenditure on solar & wind

	CAPEX to solar & wind power plants (ln)					
	NZT target=<2050			NZT target =<2045		
	(1)	(2)	(3)	(4)	(5)	(6)
NZTStatus1	-0.188	-0.232	-0.240			
	(-1.10)	(-1.64)	(-1.61)			
**NZTStatus2**				**0.635*** **	**0.496*** **	**0.637*** **
				**(5.39)**	**(4.63)**	**(5.66)**
EPS	-0.162	0.158	0.146	-0.276	0.096	0.081
	(-0.69)	(0.99)	(0.72)	(-1.27)	(0.59)	(0.42)
GDP per capita	0.000**	0.000*	0.000	0.000*	0.000	0.000
	(2.24)	(1.88)	(1.64)	(1.91)	(1.71)	(1.48)
MV	0.159**	0.159**	0.099	0.160**	0.163**	0.102*
	(2.56)	(2.48)	(1.69)	(2.60)	(2.58)	(1.77)
Profit margin	0.122**	0.116**	0.147***	0.118**	0.111**	0.141**
	(2.52)	(2.29)	(2.93)	(2.44)	(2.15)	(2.72)
Leverage	-0.057**	-0.059**	-0.058**	-0.055**	-0.056**	-0.055**
	(-2.36)	(-2.43)	(-2.65)	(-2.29)	(-2.38)	(-2.51)
Country FE	YES	YES	YES	YES	YES	YES
Year FE	NO	YES	YES	NO	YES	YES
Energy type FE	NO	NO	YES	NO	NO	YES
Observation	5179	5179	5179	5179	5179	5179
adj. R-sq	0.138	0.154	0.230	0.137	0.154	0.230

Note: This table reports the results relating corporate investments in solar & wind power plants to the enactment of NZT Laws. Specifically, we estimate the OLS model of the logarithm of capital expenditure on an indicator variable of NZTStatus. It comprises two dummy variables that represent the different statuses of the national NZT legislation. NZTStatus1 in Columns (1) to (3) equals one if the national NZT law has been passed by year t in country c, and the NZT target is set for 2050 or earlier. NZTStatus2 in Columns (4) to (6) equals one if the national NZT law has been passed by year t in country c, and the NZT target is set for 2045 and earlier. EPS is the Environmental Policy Stringency Index, downloaded from the OECD database. Plant-level data are sourced from Rystad. We also control for GDP per capita and firm-level characteristics, including market value, profit margin, and leverage. All regressions are estimated with year, country, and energy type fixed effects, and the standard errors are clustered at the country level. Data is for the period 2014 to 2023. ***, ** and * denote significance at the 1%, 5%, and 10% levels respectively.

Table S17. National NZT legislation and capital expenditure on power plants: additional variables analysis

	CAPEX to power plants (ln)			
	NZT target=<2050		NZT target=<2045	
	(1)	(2)	(3)	(4)
NZTStatus1*LowCarbon	0.061	0.060		
	(0.11)	(0.11)		
**NZTStatus2*LowCarbon**			**0.528*** **	**0.986*** **
			**(3.54)**	**(6.60)**
NZT Status1	-0.230	-0.229		
	(-0.44)	(-0.43)		
EPS	0.119	0.120	0.083	0.083
	(0.69)	(0.69)	(0.53)	(0.53)
GDP per capita	0.000*	0.000*	0.000*	0.000*
	(2.04)	(2.04)	(1.88)	(1.88)
log(MV)	0.096	0.096	0.098	0.098
	(1.59)	(1.58)	(1.62)	(1.62)
Profit margin	0.143***	0.143***	0.139***	0.139***
	(3.13)	(3.13)	(3.00)	(3.00)
Leverage	-0.046**	-0.046**	-0.044**	-0.044**
	(-2.47)	(-2.47)	(-2.30)	(-2.30)
Electricity use	1.042	1.040	1.633	1.631
	(0.60)	(0.60)	(0.98)	(0.98)
Oil price	="	0.019	="	0.018
	="	(1.14)	="	(1.06)
Country FE	YES	YES	YES	YES
Year FE	YES	YES	YES	YES
Energy FE	YES	YES	YES	YES
Company FE	NO	YES	NO	YES
Observation	5956	5956	5956	5956
adj. R-sq	0.310	0.310	0.311	0.310

Note: This table reports the results relating corporate power plant investments to the enactment of NZT Laws. Specifically, we estimate the OLS model of the number of power plants by energy type that a firm invests in during a year on an indicator variable of NZTStatus*LowCarbon. NZTStatus comprises two dummy variables that represent the different statuses of the national NZT legislation. NZTStatus1 in Columns (1) to (3) equals one if the national NZT law has been passed by year t in country c, and the NZT target is set for 2050 or earlier. NZTStatus2 in Columns (4) to (6) equals one if the national NZT law has been passed by year t in country c, and the NZT target is set for 2045 or earlier. LowCarbon is a dummy variable and equals one if the project involves solar, wind, hydro, geothermal, battery, or storage technologies. EPS is the Environmental Policy Stringency Index, downloaded from the OECD database. Plant-level data are sourced from Rystad. We also control for GDP per capita and firm-level characteristics, including market value, profit margin, and leverage. Additionally, we include country-level variables of energy use and firm-level return on assets in all regressions. We also include firm fixed effect in additional to year, country, and energy type fixed effects in columns (2) and (4). The standard errors are clustered at the country level. Data is for the period 2014 to 2023. ***, ** and * denote significance at the 1%, 5%, and 10% levels respectively.

Table S18. National NZT legislation and capital expenditure on low carbon power plants: additional variables analysis

	CAPEX to low carbon power plants (ln)			
	NZT target=<2050		NZT target=<2045	
	(1)	(2)	(3)	(4)
NZTStatus1	-0.200	-0.200		
	(-1.34)	(-1.34)		
NZTStatus2			0.683***	0.683***
			(6.17)	(6.17)
EPS	0.160	0.161	0.111	0.111
	(0.85)	(0.85)	(0.67)	(0.67)
GDP per capita	0.000*	0.000*	0.000*	0.000*
	(1.93)	(1.93)	(1.75)	(1.75)
log(MV)	0.094	0.094	0.097	0.097
	(1.55)	(1.54)	(1.59)	(1.59)
Profit margin	0.152***	0.152***	0.147***	0.147***
	(3.31)	(3.31)	(3.14)	(3.14)
Leverage	-0.052**	-0.052**	-0.050**	-0.050**
	(-2.77)	(-2.77)	(-2.63)	(-2.63)
Electricity use	-0.655	-0.656	-1.545	-1.546
	(-0.14)	(-0.14)	(-0.34)	(-0.34)
Oil price		0.019		0.017
		(0.92)		(0.81)
Country FE	YES	YES	YES	YES
Year FE	YES	YES	YES	YES
Energy type FE	YES	YES	YES	YES
Company FE	NO	YES	NO	YES
Observation	5776	5776	5776	5776
adj. R-sq	0.251	0.251	0.251	0.251

Note: This table reports the results relating corporate investments in low carbon power plants to the enactment of NZT Laws. Specifically, we estimate the OLS model of the number of power plants by energy type that a firm invests in during a year on an indicator variable of NZTStatus*LowCarbon. NZTStatus comprises two dummy variables that represent the different statuses of the national NZT legislation. NZTStatus1 in Columns (1) to (3) equals one if the national NZT law has been passed by year t in country c, and the NZT target is set for 2050 or earlier. NZTStatus2 in Columns (4) to (6) equals one if the national NZT law has been passed by year t in country c, and the NZT target is set for 2045 or earlier. LowCarbon is a dummy variable and equals one if the project involves solar, wind, hydro, geothermal, battery, or storage technologies. EPS is the Environmental Policy Stringency Index, downloaded from the OECD database. Plant-level data are sourced from Rystad. We also control for GDP per capita and firm-level characteristics, including market value, profit margin, and leverage. Additionally, we include country-level variables of energy use and firm-level return on assets in all regressions. We also include firm fixed effect in additional to year, country, and energy type fixed effects in columns (2) and (4). The standard errors are clustered at the country level. Data is for the period 2014 to 2023. ***, ** and * denote significance at the 1%, 5%, and 10% levels respectively.

Table S19. National NZT legislation and corporate NZT disclosure

	Firm NZT all	Firm NZT target	Firm NZT Finance	Firm NZT policy	Firm NZT gov	Firm NZT all	Firm NZT target	Firm NZT Finance	Firm NZT policy	Firm NZT gov
	National net zero target=<2050					National net zero target<=2045				
	(1)	(2)	(3)	(4)	(5)	(6)	(7)	(8)	(9)	(10)
NZTStatus1	0.302	0.410	0.090	-0.245	0.047					
	(0.61)	(1.52)	(0.95)	(-1.27)	(0.28)					
**NZTStatus2**						2.079***	0.979***	0.508***	0.185	0.408
						(4.51)	(4.05)	(4.52)	(1.09)	(1.36)
EPS	-0.469	-0.388	-0.120	-0.012	0.051	-0.402	-0.297	-0.101	-0.066	0.061
	(-0.81)	(-0.67)	(-1.50)	(-0.04)	(0.18)	(-0.74)	(-0.52)	(-1.54)	(-0.17)	(0.21)
GDP per capita	-0.000	0.000	-0.000*	-0.000	-0.000	-0.000	0.000	-0.000**	-0.000	-0.000
	(-1.24)	(0.67)	(-1.75)	(-1.29)	(-1.51)	(-1.26)	(0.45)	(-2.26)	(-1.03)	(-1.53)
MV	1.198***	0.245***	0.132***	0.318***	0.502***	1.191***	0.238***	0.131***	0.322***	0.501***
	(11.24)	(4.37)	(4.74)	(10.61)	(15.02)	(11.32)	(4.09)	(4.80)	(10.14)	(15.25)
Profit margin	-0.281	-0.026	-0.056*	-0.096	-0.103	-0.278	-0.020	-0.055*	-0.100	-0.103
	(-1.54)	(-0.74)	(-1.91)	(-1.33)	(-1.48)	(-1.53)	(-0.56)	(-1.88)	(-1.38)	(-1.47)
Leverage	-0.015	-0.003	-0.031	-0.008	0.027	-0.015	-0.005	-0.031	-0.006	0.027
	(-0.28)	(-0.21)	(-1.15)	(-0.79)	(1.45)	(-0.26)	(-0.33)	(-1.16)	(-0.61)	(1.45)
Year FE	YES	YES	YES	YES	YES	YES	YES	YES	YES	YES
Country FE	YES	YES	YES	YES	YES	YES	YES	YES	YES	YES
Observation	5960	5960	5960	5960	5960	5960	5960	5960	5960	5960
adj. R-sq	0.686	0.524	0.363	0.549	0.639	0.687	0.522	0.365	0.545	0.639

Note: This table reports the results relating corporate NZT disclosure to the passing of national NZT laws. The dependent variable is the corporate NZT disclosure score. We decompose the overall NZT score into NZT target and strategy, NZT finance, NZT policy and NZT governance. NZTStatus comprises two dummy variables that represent the different statuses of the national NZT legislation. NZTStatus1 in Columns (1) to (5) equals one if the national NZT law has been passed by year t in country c, and the NZT target is set for 2050 or earlier. NZTStatus2 in Columns (6) to (10) equals one if the national NZT law has been passed by year t in country c, and the NZT target is set for 2045 or earlier. EPS is the environmental policy stringency index downloaded from the OECD database. Plant-level data are sourced from Rystad. We also control for GDP per capita and firm-level characteristics, including market value, profit margin, and leverage. All regressions are estimated with year, country, and energy type fixed effects, and the standard errors are clustered at the country level. Data is for the period 2014 to 2023. ***, ** and * denote significance at the 1%, 5%, and 10% levels respectively.

Table S20. National NZT legislation and capital expenditure on low carbon power plants, interaction with corporate NZT disclosure

	CAPEX to low carbon power plants (ln)									
	National net zero target=<2050					National net zero target<=2045				
	(1)	(2)	(3)	(4)	(5)	(6)	(7)	(8)	(9)	(10)
NZTStatus1*Firm_NZT_all	-0.023									
	(-0.61)									
NZTStatus1*Firm_NZT_Tar		**-0.114**								
		**(-1.51)**								
NZTStatus1*Firm_NZT_Fin			-0.049							
			(-0.25)							
NZTStatus1*Firm_NZT_Pol				-0.137						
				(-0.61)						
NZTStatus1*Firm_NZT_Gov					-0.015					
					(-0.18)					
NZTStatus2*firm_NZT						0.061**				
						(2.22)				
NZTStatus2*firm_NZT_Tar							0.152*			
							(1.89)			
NZTStatus2*firm_NZT_Fin								0.574***		
								(5.33)		
NZTStatus2*firm_NZT_Pol									0.320*	
									(1.78)	
NZTStatus2*firm_NZT_Gov										0.102
										(1.39)
NZTStatus1	0.044	0.084	-0.093	0.071	-0.093					
	(0.17)	(0.47)	(-0.53)	(0.20)	(-0.32)					
NZTStatus2						-0.038	0.158	-0.270	-0.048	0.103
						(-0.19)	(0.99)	(-1.59)	(-0.19)	(0.45)
Controls	YES	YES	YES	YES	YES	YES	YES	YES	YES	YES
Year FE	YES	YES	YES	YES	YES	YES	YES	YES	YES	YES
Country FE	YES	YES	YES	YES	YES	YES	YES	YES	YES	YES
Observation	5776	5776	5776	5776	5776	5776	5776	5776	5776	5776
adj. R-sq	0.153	0.153	0.157	0.153	0.152	0.153	0.152	0.158	0.152	0.152

Note: This table estimates the OLS regression model of corporate investment in low carbon power plants on an indicator variable NZTStatus and on its interaction with corporate NZT disclosure score. We decompose the overall NZT score into NZT target and strategy, NZT finance, NZT policy and NZT governance. NZTStatus comprises two dummy variables that represent the different statuses of the national NZT legislation. NZTStatus1 in Columns (1) to (5) equals one if the national NZT law has been passed by year t in country c, and the NZT target is set for 2050 or earlier. NZTStatus2 in Columns (6) to (10) equals one if the national NZT law has been passed by year t in country c, and the NZT target is set for 2045 or earlier. EPS is the environmental policy stringency index downloaded from the OECD database. Plant-level data are sourced from Rystad. We also control for GDP per capita and firm-level characteristics, including market value, profit margin, and leverage. All regressions are estimated with year, country, and energy type fixed effects, and the standard errors are clustered at the country level. Data is for the period from 2014 to 2023. ***, ** and * denote significance at the 1%, 5%, and 10% levels respectively.

Table S21. National NZT legislation and capital expenditure on low carbon power plants, interaction with national NZT disclosure

	CAPEX to low carbon power plants (ln)	
	NZT target=<2050	NZT target=<2045
	(1)	(2)
NZTStatus1*Reporting	1.220***	
	(7.45)	
NZTStatus2*Reporting		0.454***
		(6.47)
NZT Status1	-0.207	
	(-1.37)	
NZT Status2		0.651***
		(6.56)
EPS	0.164	0.116
	(0.88)	(0.69)
GDP per capita	0.000*	0.000*
	(2.02)	(1.84)
log(MV)	0.094	0.097
	(1.55)	(1.60)
Profit margin	0.152***	0.147***
	(3.32)	(3.15)
Leverage	-0.052**	-0.050**
	(-2.77)	(-2.63)
Country FE	YES	YES
Year FE	YES	YES
Observation	5776	5776
adj. R-sq	0.251	0.251

Note: This table estimates the OLS regression model of corporate investment in low carbon power plants on an indicator variable NZTStatus and on its interaction with national NZT Reporting. National NZT reporting is a dummy variable measuring the reporting mechanism. It equals one if it is less than annual reporting and zero annual reporting. NZTStatus comprises two dummy variables that represent the different statuses of the national NZT legislation. NZTStatus1 in Columns (1) equals one if the national NZT law has been passed by year t in country c, and the NZT target is set for 2050 or earlier. NZTStatus2 in Columns (2) equals one if the national NZT law has been passed by year t in country c, and the NZT target is set for 2045 or earlier. EPS is the environmental policy stringency index downloaded from the OECD database. Plant-level data are sourced from Rystad. We also control for GDP per capita and firm-level characteristics, including market value, profit margin, and leverage. All regressions are estimated with year, country, and energy type fixed effects, and the standard errors are clustered at the country level. Data is for the period 2014 to 2023. ***, ** and * denote significance at the 1%, 5%, and 10% levels respectively.

Table S22. Leave one country out robustness test of national NZT legislation and capital expenditure on power plants

	CAPEX to power plants (ln)					
	NZT target=<2050			NZT target =<2045		
	Exclude DEU	Exclude FIN	Exclude AUT	Exclude DEU	Exclude FIN	Exclude AUT
	(1)	(2)	(3)	(4)	(5)	(6)
NZTStatus1*LowCarbon	0.042	0.059	0.058			
	(0.07)	(0.10)	(0.10)			
**NZTStatus2*LowCarbon**				**0.945*** **	**0.699*** **	**0.678*** **
				**(6.16)**	**(6.82)**	**(7.24)**
Controls	YES	YES	YES	YES	YES	YES
Country fixed effect	YES	YES	YES	YES	YES	YES
Year fixed effect	YES	YES	YES	YES	YES	YES
Energy type fixed effect	YES	YES	YES	YES	YES	YES
Observation	5758	5758	5935	5935	5945	5945
adj. R-sq	0.312	0.312	0.310	0.310	0.310	0.311

Notes: This table reports leave-one-country-out robustness tests of the baseline regression reported in Table 2. The subgroup of countries with statutory NZT targets of 2045 or earlier comprises Germany, Finland, and Austria. We therefore re-estimate the baseline specification after sequentially excluding each of these treatment countries. Columns (1)–(3) report results for NZTStatus1, which equals one if the national NZT law has been enacted by year t in country c and the NZT target year is 2050 or earlier. Columns (4)–(6) report results for NZTStatus2, which equals one if the national NZT law has been enacted by year t in country c and the NZT target year is 2045 or earlier. LowCarbon is a dummy variable equal to one if the project involves solar, wind, hydro, geothermal, battery, or storage technologies. All regressions include GDP per capita, environmental policy stringency, market value, profit margin, and leverage as control variables, together with country, year, and energy-type fixed effects. Standard errors are clustered at the country level. Data cover the period 2014–2023. The persistence of a positive and statistically significant coefficient after excluding each country individually indicates that the estimated effect is not driven by any single treatment country. ***, ** and * denote significance at the 1%, 5%, and 10% levels, respectively.

Table S23. National NZT legislation and capital expenditure: controlling for the existing level of renewable electricity generation

	CAPEX to power plants (ln)	
	NZT target=<2050	NZT target=<2045
	(1)	(4)
NZTStatus1*low carbon	0.058	
	(0.10)	
**NZTStatus2*low carbon**		0.722***
		(7.76)
EPS	0.125	0.088
	(0.84)	(0.69)
GDP per capita	0.000*	0.000*
	(1.97)	(1.84)
Renewable electricity	-2.542**	-2.568***
	(-2.46)	(-2.81)
log(MV)	0.095	0.097
	(1.57)	(1.61)
Profit margin	0.139***	0.135***
	(3.18)	(3.05)
Leverage	-0.050**	-0.047**
	(-2.66)	(-2.47)
Country fixed effect	YES	YES
Year fixed effect	YES	YES
Observation	5956	5956
adj. R-sq	0.311	0.312

Notes: The dependent variable is in natural logarithms. NZTStatus1 (NZTStatus2) equals one if the national NZT law has been passed by year t in country c, and the NZT target is set for 2050 (2045) or earlier. NZTStatus1 captures the average association between all statutory NZT targets of 2050 or earlier and low-carbon investment, whereas NZTStatus2 isolates the subset of countries with the most ambitious statutory targets (2045 or earlier). The two indicators are estimated separately and should not be interpreted as mutually exclusive treatment categories. LowCarbon equals one if the asset involves solar, wind, hydro, geothermal, battery, or storage technologies. Renewable electricity generation denotes the natural logarithm of annual country-level renewable electricity generation (measured in TWh). Standard errors (SEs) in parentheses are clustered at the country level. ***, ** and * denote significance at the 1%, 5%, and 10% levels respectively. CAPEX: capital expenditure; FE: fixed effect.

Method S1. Estimation of plant-level capital expenditures

To ensure robust and comparable estimates for Capex across technologies and regions over time, we applied an alternative method to estimate Capex for all power plants by energy type, continent, and first production year in our sample. All data points were extracted from the International Energy Agency's (IEA) World Energy Outlook (WEO) annual reports and other reliable data sources.

The IEA data cover a wide range of power generation technologies including coal, natural gas, nuclear, hydropower, solar, wind, biomass, and other renewables. For each technology type, we extracted relevant Capex figures (expressed in USD per kW) corresponding to the four reference years, namely 2000, 2010, 2020 and projected figures for 2025.

They are also geographically segmented into continents, aligning with standard geographical definitions: Africa, Asia, Europe, North America, South America, and Oceania. Country-level data was aggregated at the continental level by weighting each country's installed capacity to ensure representative continental averages.

To ensure consistency, we grouped power plants by energy type and continent and further segmented them into specific time periods: pre-2000, 2000-2010, 2010-2020, and post-2020. The total Capex for each power plant was calculated by multiplying the region-specific unit Capex (USD/kW) by the installed or projected installed capacities (MW) for each technology and continent in each year. Where direct historical or projected unit costs were unavailable, linear interpolation between reference years was applied to reflect historical and anticipated trends in global energy investment.

As the Rystad data only specify the first production year, we estimate the length of construction period based on the IEA data[1]. For fossil fuel power plants, we assume that they take four years from construction to production no later than 2016 and three years after 2016. For renewable power plants, we assume that they take three years to construct no later than 2016 and two years after 2016. For offshore wind plants, we assume that they take four years to build over the data sample period. We then moved our Capex estimates forward to the first construction year we estimated, as companies need to invest in advance and pay for high fixed costs upfront before power plants start to operate and generate electricity.

Based on the total Capex for each power plant, we estimate the Capex of each company weighted by its owned capacity, as some power plants are jointly owned by several companies.

Simply speaking, to estimate Capex for all power plants, we grouped power plants by energy type and continent and further segmented their first production year into specific time periods: pre-2000, 2000-2010, 2010-2020, and post-2020. The total Capex for each power plant was calculated by multiplying the region-specific unit Capex (USD/kW) by the installed or projected installed capacities (MW) for each technology and continent in each year. Where direct historical or projected unit costs were unavailable, linear interpolation between reference years was applied to reflect historical and anticipated trends in global energy investment. As the Rystad data only specify the first production year, we estimate the length of construction period based on the IEA data. Specifically, for fossil fuel power plants, we assume that they take four years from construction to production no later than 2016 and three years after 2016; For renewable power plants, we assume that they take three years to construct no later than 2016 and two years after 2016. For offshore wind plants, we assume that they take four years to build over the data sample period. We then moved our Capex estimates forward to the first construction year we estimated, as companies need to invest in advance and pay for high fixed costs upfront before power plants start to operate and generate electricity. Based on the total Capex for each power plant, we estimate the Capex of each company weighted by its owned capacity, as some power plants are jointly owned by several companies.

[1] See average power generation construction time (capacity weighted), 2010-2018, https://www.iea.org/data-and-statistics/charts/average-power-generation-construction-time-capacity-weighted-2010-2018
